# Neurological management and outcome measures in Fabry disease: consensus statements from the Italian Fabry disease neurological working group

**DOI:** 10.1186/s13023-026-04361-y

**Published:** 2026-04-24

**Authors:** Michelangelo Mancuso, Alessandro Burlina, Leonardo Ulivi, Sirio Cocozza, Anna Bersano, Mirco Cosottini, Vincenzo Donadio, Antonello Giordano, Rocco Liguori, Renzo Manara, Anna Pichiecchio, Ilaria Romani, Antonio Siniscalchi, Maurizio Tenuta, Vittoria Cianci, Antonino Tuttolomondo

**Affiliations:** 1 Department of Clinical and Experimental Medicine, Neurological Institute, University of Pisa, Pisa, Italy; 2 Department of Medicine, Neurology Unit, San Bassiano Hospital, Bassano del Grappa, Italy; 3 Division of Neurology Unit, Versilia Hospital, Lido di Camaiore, Lucca, Italy; 4 Department of Advanced Biomedical Sciences, University of Naples “Federico II”, Naples, Italy; 5 Cerebrovascular Unit Fondazione IRCCS Istituto Neurologico Carlo Besta, Milan, Italy; 6 Neuroradiology Unit, Department of Translational Research on New Technologies in Medicine and Surgery, University of Pisa, Pisa, Italy; 7 UOC Clinica Neurologica, Bellaria Hospital, IRCCS Institute of Neurological Sciences of Bologna, Bologna, Italy; 8 Dipartimento Di Scienze Biomediche E Neuromotorie, Università Degli Studi di Bologna, Bologna, Italy; 9 Neurology and Stroke Unit, P.O Guzzardi, ASP Ragusa, Vittoria, Italy; 10 Centro Clinico NeMO, IRCCS Istituto Delle Scienze Neurologiche di Bologna, Bologna, Italy; 11 Neuroradiology Unit, Department of Neurosciences, University Hospital of Padova, Padova, Italy; 12 Department of Brain and Behavioral Sciences, University of Pavia, Pavia, Italy; 13 Neuroradiology Department, IRCCS Mondino Foundation, Pavia, Italy; 14 Department of Neurosciences, Psychology, Pharmacology and Child Health, University of Florence, 50139 Firenze, Italy; 15 Department of Neurology and Stroke Unit, Annunziata Hospital of Cosenza, Cosenza, Italy; 16 Neurology Unit, University Hospital “San Giovanni di Dio e Ruggi d’Aragona”, 84131 Salerno, Italy; 17 Neurology Unit, Great Metropolitan “Bianchi-Melacrino-Morelli” Hospital, Reggio Calabria, Italy; 18 Department of Health Promotion, Mother and Child Care, Internal Medicine and Medical Specialties (ProMISE), Università degli Studi di Palermo, Piazza delle Cliniche 2, 90127 Palermo, Italy; 19 Internal Medicine and Stroke Care Ward, University Hospital, Policlinico “P. Giaccone”, 90100 Palermo, Italy

**Keywords:** Fabry disease, Neurological involvement, Neuroimaging, Outcome measures, PROMs, Clinical trials

## Abstract

**Background:**

Fabry disease (FD) is a rare X-linked lysosomal storage disorder with significant neurological involvement affecting the central, peripheral, and autonomic nervous systems. Despite its clinical burden, clear guidelines or clinical tools for the neurological assessment and monitoring of FD remain limited. Aim of the present work is to develop expert consensus recommendations on neurological management and outcome measures in FD, including the use of clinical scales, patient-reported outcome measures (PROMs), neuroimaging, and emerging digital health technologies (DHTs).

**Methods:**

A modified Delphi approach was conducted by the Italian Fabry Disease Neurological Working Group. Neurologists and neuroradiologists from Italian centers of excellence reviewed the literature, developed key statements, and participated in structured anonymous voting. The process followed European guidelines for rare diseases and included both pre-meeting surveys and an in-person consensus workshop.

**Results:**

Strong consensus was reached on a comprehensive set of clinical, functional, and patient-reported outcome measures for assessing neurological involvement in FD across age groups. Recommendations were made for brain imaging protocols, cognitive screening tools, pain and autonomic function assessments. Several clinical tools developed for related monogenic small vessel diseases may be applicable, but their use in FD requires further validation. No validated DHTs for FD assessment currently exist, and their integration into clinical research requires further investigation.

**Conclusions:**

These consensus-based recommendations represent the first systematic effort to standardize neurological outcome measures in FD. They aim to support clinical monitoring and guide future clinical trials, while highlighting the need for prospective validation studies and development of FD-specific digital tools.

**Supplementary Information:**

The online version contains supplementary material available at 10.1186/s13023-026-04361-y.

## Introduction

Fabry disease (FD) is an inherited x-linked lysosomal storage disorder caused by a deficiency or absence of the enzyme α-galactosidase A, due to over 1000 pathogenic variants in the *GLA* gene (OMIM#301500) [1]. Affected males typically develop the classic, severe phenotype, while heterozygous females can exhibit a wide range of clinical manifestations, from asymptomatic to severe involvement of vital organs [2]. The classic form of FD presents with early-onset signs and symptoms, whereas later-onset variants may manifest clinically later in life.

As a result of the enzyme deficiency, the sphingolipid globotriaosylceramide (GL-3 or Gb3) and its deacetylated derivative, lysoglobotriaosylceramide (lyso-GL-3 or lyso-Gb3), accumulate in a wide range of cell types, including vascular endothelial and smooth muscle cells, cardiomyocytes, renal tubular cells, enteric neurons, and peripheral nerve fibers. This accumulation leads to multi-organ involvement, most commonly affecting the heart, kidneys, brain, peripheral nervous system, and gastrointestinal tract [2].

Neurological manifestations of FD are diverse, involving the central, peripheral, and autonomic nervous systems. Common features include small fiber neuropathy leading to pain and dysesthesia; cerebrovascular complications such as early-onset stroke and white matter lesions; cognitive impairment and psychiatric signs; and autonomic symptoms such as hypohidrosis and gastrointestinal dysmotility [3–7]. These manifestations can significantly impair quality of life and may occur even in early stages of the disease.

Despite the significant impact of FD on the nervous system, clear guidance on neurological monitoring remains limited. Apart from an expert panel which has focused on small fiber neuropathy and neuropathic pain [8], there are currently no standardized recommendations for assessing neurological involvement in FD or for selecting appropriate outcome measures to monitor disease progression and treatment response. This contrasts with the renal and cardiac domains, where both clinical guidelines and consensus statements have been well established [9, 10].

To address this gap, a panel of expert neurologists and neuroradiologists implemented a rigorous modified Delphi process with the following objectives:

- to review current knowledge and literature on neurological involvement in FD;
- to evaluate available neurological outcome measures (OMs) and patient-reported outcome measures (PROMs);
- to achieve consensus on an optimal set of OMs, PROMs and neuroimaging biomarkers, and indicators of disease burden suitable for use in natural history studies and clinical trials;
- to explore the potential role of emerging digital health technologies (DHTs) in this field.

## Preparatory work and methodology

The Delphi method was employed to develop this consensus. This methodology offers a structured approach for gathering expert opinions (the “Delphi Panel”) and has been widely used to achieve consensus on clearly defined topics, including various aspects of FD [3, 10, 11]. Although referred to as a “panel”, experts participated independently, providing their input freely and voting anonymously. Delphi process included three phases: pre-meeting survey, in-person workshop with iterative voting, and post-meeting confirmation of the final statements. To ensure methodological rigor, particularly in the context of a rare disease such FD, recently published supporting tools were also followed: the European Reference Network Clinical Practice Guidelines and Clinical Decision Support Tools (available at https://www.erknet.org/fileadmin/files/user_upload/0._Intro_Toolkit__D-B.2_.pdf), and the European Academy of Neurology guidance for developing and reporting clinical practice guidelines on rare neurological diseases [12]. In addition, the study was reported in accordance with the ACCORD (ACcurate COnsensus Reporting Document) checklist to ensure transparent and comprehensive reporting of the consensus process [13].

### Phase I: Pre-meeting phase

The Italian Fabry Disease Neurological Working Group, co-chaired by MM, VC, and AT, invited Italian experts from recognized centers of excellence based on their established expertise in FD. To ensure a diverse panel, invitees were encouraged to recommend additional participants to achieve balanced representation in terms of geography and gender. Invitations were sent via email, outlining the study objectives and the Delphi methodology.

The consensus panel included 16 participants from across Italy. Two experts (AP and AG), who actively contributed to the preliminary work, were unable to attend the live meeting in Pisa.

During the initial virtual meeting, participants discussed and unanimously approved the methodology to explore neurological involvement in FD adults and children above the age of 16 years, including the literature search strategy. The experts also agreed to broaden the literature search to include CADASIL and other monogenic cerebral small vessel diseases, given the limited neurological literature specific to FD and the comparable patterns of white matter involvement and neurological signs observed in these related conditions [3, 6, 14, 15]. The search included articles published from database inception to March 31, 2025, in MEDLINE via PubMed, ClinicalTrials.gov, and the European Clinical Trials Register. Only English-language publications were considered. Unpublished data, abstracts, and conference proceedings were excluded. The detailed literature search strategy, the PRISMA and selected articles are provided in Supplemental File 1. MM and LU also developed a survey (Supplementary File 2) to evaluate the level of consensus among experts regarding selected OMs, neuroimaging studies, clinical scales, and PROMs, and feedback on the results was provided at the beginning of the live meeting. The selection of clinical tools and PROMs was based on literature review, clinical relevance to FD manifestations, feasibility in practice, and prior validation in related neurological conditions. A formal systematic psychometric evaluation was not performed. Regarding PROMs—reports on a patient’s health status provided directly by the patient without interpretation by healthcare providers or others—previous data on the most bothersome symptoms of FD were reviewed to ensure inclusion of those with neurological relevance [16, 17].

Responses were collected anonymously through the SurveyMonkey platform, analysed and discussed ahead of the in-person meeting. Participants used a 5-point Likert scale [17] to express their agreement with each statement (1: absolutely disagree; 2: disagree; 3: neutral; 4: agree; 5: absolutely agree). A strong consensus was defined as > 70% of responses scoring ≥ 4 or ≤ 2, with a mean score of ≥ 4 or ≤ 2. If only one condition was met, a consensus (but not strong) was **considered ** reached. Statements failing to meet these criteria **(listed at the end of the Supplementary file ****3****)** were considered to lack consensus.

## Results

### Live meeting general considerations

At the workshop (held in Pisa, June 9th and 10th 2025), following a warm welcome and introductory remarks by the three co-organizers, the sessions began with MM presenting the results of both literature search and of the survey. This was followed by an overview of current neurological management in FD, delivered by AB.

The second half of the first day was dedicated to an in-depth discussion of both central and peripheral nervous system involvement and of neuroimaging protocols. By the end of the day, new statements had been drafted, some previous queries revised, and an anonymous voting session was conducted.

On the second day, participants reviewed and discussed the statements voted on the first day. The focus then shifted to the autonomic nervous system and the role of digital health technologies. Additional statements were formulated, previous queries were updated, and another round of anonymous voting was held. At the end of the second day, the outcomes of all voting sessions were reviewed and discussed. A few additional queries were proposed, followed by a final round of voting. The workshop concluded with unanimous agreement on the importance of continued monitoring and a shared commitment to revisiting this consensus within the next 3–4 years, if necessary. A unanimous post-meeting confirmation of the final statements drafted during the live session was also achieved.

All statements and voting results developed during the workshop are provided as Supplementary File S3.

### Consensus of measures suitable to assess FD patients both longitudinally and in clinical studies

Table 1 presents a selection of the most important statements developed during the meeting, while Table 2 reports the measures endorsed by the panellists for assessing FD in both adolescents (aged *≥* 16) and adults.

**Table 1:** Statements of the Delphi working group

Domain	Topic	Consensus Statement	Consensus Level
CNS	Brain health	The vascular risk factor profile of patients with Fabry disease should be evaluated and treated when indicated. Lifestyle changes such as stopping smoking, healthy diet and physical exercise should be advised.	Strong
CNS	Stroke	Patients should be educated about stroke symptoms and the importance of early medical intervention in case of new neurological events.	Strong
CNS	Stroke	Fibrinolysis and/or thrombectomy are not contraindicated in Fabry disease	Strong
CNS	Stroke	Secondary prevention for cerebrovascular disease in Fabry should adhere to the international guidelines (i.e. ESO and AHA-ASA).	Strong
CNS	Stroke	Patients with Fabry disease and a history of stroke should undergo neurological follow-up at least annually, including clinical examination and, if appropriate, repeat neuroimaging.	Strong
CNS	Brain Health	A comprehensive neurological assessment should be performed annually or whenever clinical progression occurs.	Strong
CNS	Cognition	Cognitive screening should be performed in all Fabry disease patients at diagnosis, regardless of symptom presentation	Strong
CNS	Cognition	Cognitive function should be reassessed every 2 years in asymptomatic patients and every 12 months in patients with prior stroke, progressive symptoms, or neurological involvement.	Strong
CNS	Mood disorders	Depression, anxiety, and other mood disorders should be routinely screened in Fabry patients with cognitive complaints, as they may contribute to cognitive symptoms.	Strong
CNS	Sleep disorders	Sleep disturbances may represent a significant concern in Fabry disease and should be actively investigated when suspected.	Strong
Neuroimaging		Brain MRI with a standardized protocol, including T1-weighted, T2-weighted, FLAIR, diffusion-weighted imaging (DWI), and T2* weighted Gradient Echo (GRE) or, if available, susceptibility-weighted imaging (SWI), should be the primary modality for the diagnosis and follow-up of neuroradiological involvement in Fabry disease.	Strong
Neuroimaging		Magnetic Resonance Angiography should be performed in all Fabry patients to detect vertebrobasilar dolichoectasia and other cerebrovascular abnormalities at the baseline.	Strong
Neuroimaging		Patients with Fabry disease should undergo brain MRI at baseline and every 3 years to monitor cerebrovascular involvement, with more frequent imaging in those with clinical progression or new neurological symptoms.	Strong
PNS	Oto vestibular involvement	Otovestibular symptoms should be investigated at the time of neurological evaluation.	Strong
PNS	Oto vestibular involvement	Audiometric examination should be performed in patients with Fabry disease at baseline and repeated every 3 years, or whenever hearing impairment (or its worsening) occurs.	Strong
PNS	Neuropathic pain	Pain questionnaires should be administered at diagnosis, every 12 months, and whenever pain management therapy occur	Strong
ANS	General considerations	Autonomic dysfunction assessment and management should be tailored to the patient’s symptoms, especially in those with autonomic small fiber neuropathy (i.e. cardiovascular dysautonomia and/or severe gastrointestinal symptoms).	Strong
ANS	GI	Gastrointestinal dysmotility requires early evaluation using validated clinical scales to optimize symptom management and improve patient quality of life.	Strong
Digital Health Tools		There are no validated digital tools for Fabry disease assessment to date, with no pivotal studies on this topic published. More studies need to be done to further develop and validate such digital tools in Fabry disease.	Strong

CNS: Central Nervous System; PNS: Peripheral Nervous System; ANS: Autonomic Nervous System; GI: gastro intestinal

**Table 2:** Measures suitable to assess patients during the disease course and in clinical studies (for both adults and children above the age of 16 years). PROM: patient reported outcome measure

Measure Type	Tools	Recommended Use
Clinical scale	The Montreal Cognitive Assessment (MoCA)	Monitoring cognitive impairment
Clinical scale	The Hospital Anxiety and Depression scale (HADS)	Monitoring mood disorders
Clinical scales	▪ The Modified Rankin Scale (mRS) ▪ Barthel Index (BI) ▪ NIHSS	To assess long-term disability
Clinical scales	▪ Würzburg Fabry Pain Questionnaire ▪ Brief Pain Inventory (BPI) ▪ Short-Form McGill Pain Questionnaire (SF-MPQ) ▪ Neuropathic Pain Symptom Inventory	Neuropathic pain assessment
Clinical scale	The Composite Autonomic Symptom Score (COMPASS31)	Overall assessment of dysautonomia
PROM	Fatigue Severity Scale	Overall assessment of fatigue
PROM	The 24-hour or 7-day Fabry disease Patient-Reported Outcome-Gastrointestinal (FABPRO-GI)	Overall assessment of gastrointestinal symptoms
PROM	The 5-level EQ-5D version (5Q-5D-5 L)	Measuring generic health-related quality of life
PROM	Fabry Disease Patient-Reported Outcome(FD-PRO)	To measure symptom severity in Fabry disease

The statements reported in Table 1 highlight several areas considered essential for the longitudinal monitoring of patients with FD. Strong consensus was reached on the importance of routine neuroimaging and vascular risk assessment, with experts recommending brain MRI using standardized protocols at baseline and every three years, or more frequently if clinically indicated. The recommended MRI protocol includes T1-weighted, T2-weighted, FLAIR, diffusion-weighted imaging (DWI), and T2* weighted Gradient Echo (GRE) or, if available, susceptibility-weighted imaging (SWI), with Magnetic Resonance Angiography at baseline. Quantitative measures of lesion burden may be considered in research settings. Imaging findings should be integrated with clinical and cognitive assessments for longitudinal monitoring. Particular attention should be given to cerebrovascular complications, including stroke risk, and adherence to international secondary prevention guidelines. Education on stroke signs and symptoms and timely intervention was also emphasized.

In addition, the panel endorsed annual comprehensive neurological evaluations and regular screening for cognitive decline, psychiatric symptoms, and -when indicated- sleep disturbances. Cognitive function and mood should be systematically reassessed based on individual risk profiles, with the Montreal Cognitive Assessment (MoCA) and the Hospital Anxiety and Depression Scale (HADS) were favoured tools for routine screening (Table 2). Experts also recognized the relevance of peripheral and autonomic nervous system involvement, recommending validated examinations such as skin biopsy [18], Quantitative Sensory Testing (QST) [19], sympathetic skin response [20] and clinical scales such as the Würzburg Fabry Pain Questionnaire and COMPASS-31.

Although several clinical scales have been proposed as potentially useful in FD, most have neither been validated nor specifically applied to this disease. Their recommendation was based primarily on their relevance in other monogenic small vessel diseases, particularly CADASIL [3]. The experts emphasized the need for prospective studies in FD to assess the appropriateness and effectiveness of these scales, OMs, and PROMs in addressing the unique features of the disease.

Finally, following an in-depth discussion on the potential role of DHTs, the panel agreed that no validated DHTs currently exist for the assessment of FD, and no pivotal studies on this topic have been published to date. Further research is required to develop and validate DHTs tailored to the specific needs of FD patients.

## Discussion

In this consensus, experts from Italy met to discuss the optimal neurological management of patients with FD to assist clinicians and patients in decision-making.

This consensus represents the first systematic attempt to define standardized neurological outcome measures for FD, addressing a long-standing gap in clinical guidance. Through a structured Delphi process involving expert neurologists and neuroradiologists from across Italy, the working group developed a comprehensive set of recommendations covering central, peripheral, and autonomic nervous system involvement. These include specific clinical scales and PROMs applicable to both adults and children over the age of 16, as well as neuroimaging protocols. Emphasis was placed on validated tools that can be feasibly implemented in both routine clinical care and research settings. The endorsed measures are intended to support consistent longitudinal monitoring of FD progression, guide treatment decisions, and facilitate the design of future clinical trials with a specific neurological focus.

It is important to emphasize that while consensus-based information can offer valuable guidance, treatment decisions for the various neurological manifestations of FD must always be individualized, considering each patient’s specific needs and risk profile. Moreover, these recommendations are not evidence-based. Although consensus methods are commonly employed to guide clinical practice when empirical data are lacking, expert opinion remains low on the hierarchy of evidence. However, the recommendations presented here are the result of a consensus approach based on clinical experience and the available literature and, therefore, may be useful in improving the quality of clinical decision and patient outcome in FD.

Finally, the group acknowledged the limitations of existing tools, many of which were originally developed for other cerebrovascular conditions or other monogenic small vessel diseases and stressed the urgent need for validation studies in FD populations. The lack of disease-specific DHTs also emerged as a critical unmet need. Potential applications of DHTs include remote monitoring of pain, autonomic dysfunction, gait, and cognitive performance. Experience from other neurological rare diseases suggests these tools may improve longitudinal monitoring and enable decentralized trials [21, 22]. Further research is required to validate digital endpoints in FD populations.

In conclusion, the consensus statements developed in this Delphi process seek to address an unmet need for providing recommendations on the neurological monitoring in patients with FD. Future research should prioritize the validation and refinement of these outcome measures, the development of disease-specific digital tools, and the overall advancement of neurological care in patients with FD.

## Supplementary Information

Below is the link to the electronic supplementary material.

Supplementary Material 1

Supplementary Material 2

Supplementary Material 3

## Data Availability

The anonymized dataset used and/or analysed during the current study is available from the corresponding author on reasonable request.
