# Supplementary material for: Neurological management and outcome measures in Fabry disease: consensus statements from the Italian Fabry disease neurological working group
Source: Orphanet J Rare Dis. 2026 Apr 24;21:222. doi: 10.1186/s13023-026-04361-y (PMC13277257; doi:10.1186/s13023-026-04361-y)
Supplement: Supplementary file 1 — Supplementary Material 1 [file 13023_2026_4361_MOESM1_ESM.pdf]

SUPPLEMENTARY FILE 1

CENTRAL NERVOUS SYSTEM INVOLVEMENT IN FABRY DISEASE

A comprehensive literature search was conducted to identify relevant studies on stroke and cognitive decline in Fabry disease. The search strategy utilized Medical Subject Headings (MeSH) and keyword terms to capture a broad yet targeted range of studies. The search was structured into two main components: (1) Fabry disease and related small vessel diseases and (2) stroke, cognitive dysfunction, and neuropsychiatric outcomes. Specifically, Fabry disease was searched using MeSH terms and synonyms, including “Fabry Disease”, “Anderson-Fabry Disease” and comparisons with other cerebral small vessel diseases like CADASIL and cerebral small vessel disease. The second component included stroke-related terms such as “Stroke”, “Cerebrovascular Disorders, Brain Ischemia”, and “Lacunar Stroke” alongside cognitive and neuropsychiatric terms like “Cognitive Dysfunction”, “Cognitive Impairment”, Dementia”, “Memory Disorders”, “Executive Function”, “Mood Disorders”, “Depression”, “Anxiety Disorders” and “Psychiatric Disorders”. The search was conducted in major biomedical databases, ensuring comprehensive coverage of peer-reviewed studies relevant to the topic.

PRISMA

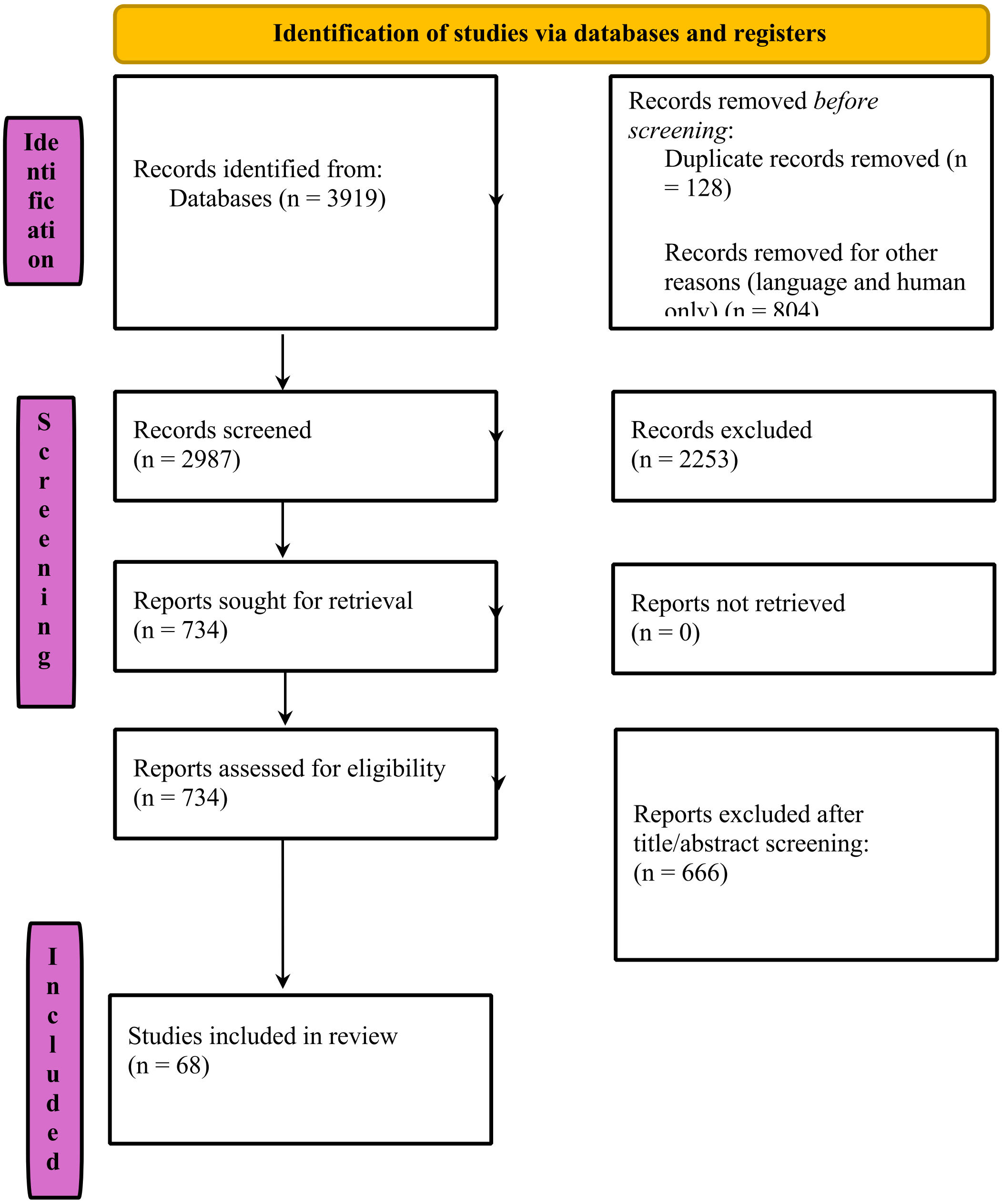

REFERENCE

1. Bolsover FE, Murphy E, Cipolotti L, Werring DJ, Lachmann RH. Cognitive dysfunction and depression in Fabry disease: a systematic review. J Inherit Metab Dis. 2014 Mar;37(2):177-87. doi: 10.1007/s10545-013-9643-x. Epub 2013 Aug 16. PMID: 23949010.
2. Murphy P, Williams F, Davagnanam I, Chan E, Murphy E, Hughes D, Quattrocchi G, Werring DJ, Lachman RH, Cipolotti L. Cognitive dysfunction and white matter hyperintensities in Fabry disease. J Inherit Metab Dis. 2022 Jul;45(4):782-795. doi: 10.1002/jimd.12472. Epub 2022 Apr 2. PMID: 34994980.
3. Körver S, Geurtsen GJ, Hollak CEM, van Schaik IN, Longo MGF, Lima MR, Dijkgraaf MGW, Langeveld M. Cognitive functioning and depressive symptoms in Fabry disease: A follow-up study. J Inherit Metab Dis. 2020 Sep;43(5):1070-1081. doi: 10.1002/jimd.12271. Epub 2020 Jun 25. PMID: 32510623; PMCID: PMC7540266.

4. Körver S, Geurtsen GJ, Hollak CEM, van Schaik IN, Longo MGF, Lima MR, Vedolin L, Dijkgraaf MGW, Langeveld M. Predictors of objective cognitive impairment and subjective cognitive complaints in patients with Fabry disease. Sci Rep. 2019 Jan 17;9(1):188. doi: 10.1038/s41598-018-37320-0. PMID: 30655570; PMCID: PMC6336934.

5. Körver S, van de Schraaf SAJ, Geurtsen GJ, Hollak CEM, van Schaik IN, Langeveld M. The Mini Mental State Examination does not accurately screen for objective cognitive impairment in Fabry Disease. JIMD Rep. 2019 May 20;48(1):53-59. doi: 10.1002/jmd2.12036. PMID: 31392113; PMCID: PMC6606981.

6. Körver S, Vergouwe M, Hollak CEM, van Schaik IN, Langeveld M. Development and clinical consequences of white matter lesions in Fabry disease: a systematic review. Mol Genet Metab. 2018 Nov;125(3):205-216. doi: 10.1016/j.ymgme.2018.08.014. Epub 2018 Sep 5. PMID: 30213639.

7. Körver S, Geurtsen GJ, Hollak CEM, van Schaik IN, Longo MGF, Lima MR, Vedolin L, Dijkgraaf MGW, Langeveld M. Depressive symptoms in Fabry disease: the importance of coping, subjective health perception and pain. Orphanet J Rare Dis. 2020 Jan 28;15(1):28. doi: 10.1186/s13023-020-1307-y. PMID: 31992347; PMCID: PMC6986064.

8. Sigmundsdottir L, Tchan MC, Knopman AA, Menzies GC, Batchelor J, Sillence DO. Cognitive and psychological functioning in Fabry disease. Arch Clin Neuropsychol. 2014 Nov;29(7):642-50. doi: 10.1093/arclin/acu047. PMID: 25319043; PMCID: PMC4263929.

9. Elstein D, Doniger GM, Altarescu G. Cognitive testing in Fabry disease: pilot using a brief computerized assessment tool. Isr Med Assoc J. 2012 Oct;14(10):624-8. PMID: 23193784.

10. Tuttolomondo A, Pecoraro R, Simonetta I, Miceli S, Pinto A, Licata G. Anderson-Fabry disease: a multiorgan disease. Curr Pharm Des. 2013;19(33):5974-96. doi: 10.2174/13816128113199990352. PMID: 23448451.

11. Del Tredici K, Ludolph AC, Feldengut S, Jacob C, Reichmann H, Bohl JR, Braak H. Fabry Disease With Concomitant Lewy Body Disease. J Neuropathol Exp Neurol. 2020 Apr 1;79(4):378-392. doi: 10.1093/jnen/nlz139. Erratum in: J Neuropathol Exp Neurol. 2020 Nov 1;79(11):1253. doi: 10.1093/jnen/nlaa030. PMID: 32016321; PMCID: PMC7092358.

12. Moore DF, Kaneski CR, Askari H, Schiffmann R. The cerebral vasculopathy of Fabry disease. J Neurol Sci. 2007 Jun 15;257(1-2):258-63. doi: 10.1016/j.jns.2007.01.053. Epub 2007 Mar 23. PMID: 17362993.

13. Low M, Nicholls K, Tubridy N, Hand P, Velakoulis D, Kiers L, Mitchell P, Becker G. Neurology of Fabry disease. Intern Med J. 2007 Jul;37(7):436-47. doi:10.1111/j.1445-5994.2007.01366.x. PMID: 17547722.

14. Ulivi L, Kanber B, Prados F, Davagnanam I, Merwick A, Chan E, Williams F, Hughes D, Murphy E, Lachmann RH, Wheeler-Kingshott CAMG, Cipolotti L, Werring DJ. White matter integrity correlates with cognition and disease severity in Fabry disease. Brain. 2020 Dec 5;143(11):3331-3342. doi: 10.1093/brain/awaa282.PMID: 33141169.

15. Loret G, Miatton M, Vingerhoets G, Poppe B, Hemelsoet D. A long-term neuropsychological evaluation in Fabry disease. Acta Neurol Belg. 2021 Feb;121(1):191-197. doi: 10.1007/s13760-020-01484-2. Epub 2020 Sep 11. PMID: 32915382.

16. Ohsawa I, Onuki A, Oka F, Matsuoka Y, Makita Y, Kobayashi T, Kanaguchi Y, Nakamura Y, Suzuki Y, Goto Y, Gotoh H. Rapidly progressive cognitive impairment resulting in heavy psychosocial burden in a patient with Fabry disease undergoing hemodialysis: a case report. BMC Nephrol. 2024 Jun 3;25(1):188. doi:10.1186/s12882-024-03624-9. PMID: 38831308; PMCID: PMC11149238.

17. Hamed A, DasMahapatra P, Lyn N, Gwaltney C, Hopkin RJ. Development of the Fabry Disease Patient-Reported Outcome (FD-PRO): a new instrument to measure the symptoms and impacts of Fabry Disease. Orphanet J Rare Dis. 2021 Jun 25;16(1):285. doi: 10.1186/s13023-021-01894-2. PMID: 34172077; PMCID:PMC8235809.

18. Löhle M, Hughes D, Milligan A, Richfield L, Reichmann H, Mehta A, Schapira AH. Clinical prodromes of neurodegeneration in Anderson-Fabry disease. Neurology. 2015 Apr 7;84(14):1454-64. doi: 10.1212/WNL.0000000000001450. Epub 2015 Mar 11. PMID: 25762709; PMCID: PMC4390387.

19. Lelieveld IM, Böttcher A, Hennermann JB, Beck M, Fellgiebel A. Eight-Year Follow-Up of Neuropsychiatric Symptoms and Brain Structural Changes in Fabry Disease. PLoS One. 2015 Sep 4;10(9):e0137603. doi: 10.1371/journal.pone.0137603. PMID: 26340726; PMCID: PMC4560446.

20. Sagnelli A, Savoiardo M, Marchesi C, Morandi L, Mora M, Morbin M, Farina L, Mazzeo A, Toscano A, Pagliarani S, Lucchiari S, Comi GP, Salsano E, Pareyson D. Adult polyglucosan body disease in a patient originally diagnosed with Fabry's disease. Neuromuscul Disord. 2014 Mar;24(3):272-6. doi:10.1016/j.nmd.2013.11.006. Epub 2013 Nov 19. PMID: 24380807.

21. Bugescu N, Alioto A, Segal S, Cordova M, Packman W. The neurocognitive impact of Fabry disease on pediatric patients. Am J Med Genet B Neuropsychiatr Genet. 2015 Apr;168B(3):204-10. doi: 10.1002/ajmg.b.32297. Epub 2015 Mar 4. PMID: 25739920.

22. Assareh A, Mather KA, Schofield PR, Kwok JB, Sachdev PS. The genetics of white matter lesions. CNS Neurosci Ther. 2011 Oct;17(5):525-40. doi: 10.1111/j.1755-5949.2010.00181.x. Epub 2010 Jul 7. PMID: 21951372; PMCID: PMC6493881.

23. Giau VV, Bagyinszky E, Youn YC, An SSA, Kim SY. Genetic Factors of Cerebral Small Vessel Disease and Their Potential Clinical Outcome. Int J Mol Sci. 2019 Sep 3;20(17):4298. doi: 10.3390/ijms20174298. PMID: 31484286; PMCID: PMC6747336.

24. Henke L, Ghorbani A, Mole SE. The use of nanocarriers in treating Batten disease: A systematic review. Int J Pharm. 2025 Feb 10;670:125094. doi:10.1016/j.ijpharm.2024.125094. Epub 2024 Dec 16. PMID: 39694161.

25. Segal P, Kohn Y, Pollak Y, Altarescu G, Galili-Weisstub E, Raas-Rothschild A. Psychiatric and cognitive profile in Anderson-Fabry patients: a preliminary study. J Inherit Metab Dis. 2010 Aug;33(4):429-36. doi:10.1007/s10545-010-9133-3. Epub 2010 Jun 15. PMID: 20549363.

26. Bersano A, Debette S, Zanier ER, Lanfranconi S, De Simoni MG, Zuffardi O, Micieli G. The genetics of small-vessel disease. Curr Med Chem. 2012;19(24):4124-41. doi: 10.2174/092986712802430081. PMID: 22680632.

27. Gairing S, Wiest R, Metzler S, Theodoridou A, Hoff P. Fabry's disease and psychosis: causality or coincidence? Psychopathology. 2011;44(3):201-4. doi:10.1159/000322794. Epub 2011 Mar 17. PMID: 21412034.

28. Ringelstein EB, Nabavi DG. Cerebral small vessel diseases: cerebral microangiopathies. Curr Opin Neurol. 2005 Apr;18(2):179-88. doi:10.1097/01.wco.0000162861.26971.03. PMID: 15791150.

29. Ringelstein EB, Kleffner I, Dittrich R, Kuhlenbäumer G, Ritter MA. Hereditary and non-hereditary microangiopathies in the young. An up-date. J Neurol Sci. 2010 Dec 15;299(1-2):81-5. doi: 10.1016/j.jns.2010.08.037. Epub 2010 Sep 18. PMID: 20851424.

30. Biegstraaten M, Arngrímsson R, Barbey F, Boks L, Cecchi F, Deegan PB, Feldt- Rasmussen U, Geberhiwot T, Germain DP, Hendriksz C, Hughes DA, Kantola I, Karabul N, Lavery C,

Linthorst GE, Mehta A, van de Mheen E, Oliveira JP, Parini R, Ramaswami U, Rudnicki M, Serra A, Sommer C, Sunder-Plassmann G, Svarstad E, Sweeb A, Terryn W, Tylki-Szymanska A, Tøndel C, Vujkovac B, Weidemann F, Wijburg FA, Woolfson P, Hollak CE. Recommendations for initiation and cessation of enzyme replacement therapy in patients with Fabry disease: the European Fabry Working Group consensus document. Orphanet J Rare Dis. 2015 Mar 27;10:36. doi: 10.1186/s13023-015-0253-6. PMID: 25885911; PMCID: PMC4383065.

31. Schermuly I, Müller MJ, Müller KM, Albrecht J, Keller I, Yakushev I, Beck M, Fellgiebel A. Neuropsychiatric symptoms and brain structural alterations in Fabry disease. Eur J Neurol. 2011 Feb;18(2):347-353. doi:10.1111/j.1468-1331.2010.03155.x. PMID: 20636371.

32. Cassis L, Cortès-Saladelafont E, Molero-Luis M, Yubero D, González MJ, Ormazábal A, Fons C, Jou C, Sierra C, Castejon Ponce E, Ramos F, Armstrong J, O'Callaghan MM, Casado M, Montero R, Meavilla-Olivas S, Artuch R, Barić I, Bartoloni F, Bellettato CM, Bonifazi F, Ceci A, Cvitanović-Šojat L, Dali CI, D'Avanzo F, Fumic K, Giannuzzi V, Lampe C, Scarpa M, Garcia-Cazorla Á. Review and evaluation of the methodological quality of the existing guidelines and recommendations for inherited neurometabolic disorders. Orphanet J Rare Dis. 2015 Dec 30;10:164. doi: 10.1186/s13023-015-0376-9. Erratum in: Orphanet J Rare Dis. 2016 Nov 3;11(1):147. doi: 10.1186/s13023-016-0431-1. PMID: 26714856;PMCID: PMC4696316.

33. Fellgiebel A, Wolf DO, Kolodny E, Müller MJ. Hippocampal atrophy as a surrogate of neuronal involvement in Fabry disease. J Inherit Metab Dis. 2012 Mar;35(2):363-7. doi: 10.1007/s10545-011-9390-9. Epub 2011 Sep 20. PMID: 21932096.

34. Fellgiebel A, Müller MJ, Ginsberg L. CNS manifestations of Fabry's disease. Lancet Neurol. 2006 Sep;5(9):791-5. doi: 10.1016/S1474-4422(06)70548-8. PMID:16914407.

35. Fellgiebel A. Stroke and brain structural alterations in Fabry disease. Clin Ther. 2007;29 Suppl A:S9-10. doi: 10.1016/s0149-2918(07)80118-4. Erratum in: Clin Ther. 2007 Oct;29(10):2268. PMID: 17580241.

36. Saks DG, Sachdev PS. Monogenic causes of cerebral small vessel disease-models for vascular cognitive impairment and dementia? Curr Opin Psychiatry. 2025 Mar 1;38(2):112-118. doi: 10.1097/YCO.0000000000000978. Epub 2025 Jan 22. PMID: 39840612; PMCID: PMC11789596.

37. Lenders M, Duning T, Schelleckes M, Schmitz B, Stander S, Rolfs A, Brand SM, Brand E. Multifocal white matter lesions associated with the D313Y mutation of the α-galactosidase A gene. PLoS One. 2013;8(2):e55565. doi: 10.1371/journal.pone.0055565. Epub 2013 Feb 5. PMID: 23393592; PMCID: PMC3564750.

38. Dong Y, Sharma VK, Chan BP, Venketasubramanian N, Teoh HL, Seet RC, Tanicala S, Chan YH, Chen C. The Montreal Cognitive Assessment (MoCA) is superior to the Mini-Mental State Examination (MMSE) for the detection of vascular cognitive impairment after acute stroke. J Neurol Sci. 2010 Dec 15;299(1-2):15-8. doi: 10.1016/j.jns.2010.08.051. PMID: 20889166.

39. Fu C, Jin X, Chen B, Xue F, Niu H, Guo R, Chen Z, Zheng H, Wang L, Zhang Y. Comparison of the Mini-Mental State Examination and Montreal Cognitive Assessment executive subtests in detecting post-stroke cognitive impairment. Geriatr Gerontol Int. 2017 Dec;17(12):2329-2335. doi: 10.1111/ggi.13069. Epub 2017 Jul 4. PMID: 28675607.

40. Toglia J, Fitzgerald KA, O'Dell MW, Mastrogiovanni AR, Lin CD. The Mini-Mental State Examination and Montreal Cognitive Assessment in persons with mild subacute stroke: relationship to functional outcome. Arch Phys Med Rehabil. 2011 May;92(5):792-8. doi: 10.1016/j.apmr.2010.12.034. PMID: 21530727.

41. Pendlebury ST, Cuthbertson FC, Welch SJ, Mehta Z, Rothwell PM. Underestimation of cognitive impairment by Mini-Mental State Examination versus the Montreal Cognitive Assessment in patients with transient ischemic attack and stroke: a population-based study. Stroke. 2010 Jun;41(6):1290-3. doi: 10.1161/STROKEAHA.110.579888. Epub 2010 Apr 8. PMID: 20378863.

42. Skrobot OA, Black SE, Chen C, DeCarli C, Erkinjuntti T, Ford GA, Kalaria RN, O'Brien J, Pantoni L, Pasquier F, Roman GC, Wallin A, Sachdev P, Skoog I; VICCCS group; Ben-Shlomo Y, Passmore AP, Love S, Kehoe PG. Progress toward standardized diagnosis of vascular cognitive impairment: Guidelines from the Vascular Impairment of Cognition Classification Consensus Study. Alzheimers Dement. 2018 Mar;14(3):280-292. doi: 10.1016/j.jalz.2017.09.007. Epub 2017 Oct 19. PMID: 29055812.

43. Godefroy O, Fickl A, Roussel M, Auribault C, Bugnicourt JM, Lamy C, Canaple S, Petitnicolas G. Is the Montreal Cognitive Assessment superior to the Mini-Mental State Examination to detect poststroke cognitive impairment? A study with neuropsychological evaluation. Stroke. 2011 Jun;42(6):1712-6. doi: 10.1161/STROKEAHA.110.606277. Epub 2011 Apr 7. PMID: 21474808.

44. Loeb J, Feldt-Rasmussen U, Madsen CV, Vogel A. Cognitive Impairments and Subjective Cognitive Complaints in Fabry Disease: A Nationwide Study and Review of the Literature. JIMD Rep. 2018;41:73-80. doi: 10.1007/8904_2018_103. Epub 2018 Apr 14. PMID: 29654545; PMCID: PMC6122045.

45. Sheng S, Wu L, Nalleballe K, Sharma R, Brown A, Ranabothu S, Kapoor N, Onteddu S. Fabry's disease and stroke: Effectiveness of enzyme replacement therapy (ERT) in stroke prevention, a review with meta-analysis. J Clin Neurosci. 2019 Jul;65:83-86. doi: 10.1016/j.jocn.2019.03.064. Epub 2019 Apr 4. PMID: 30955952.

46. Viana-Baptista M. Stroke and Fabry disease. J Neurol. 2012 Jun;259(6):1019-28. doi: 10.1007/s00415-011-6278-4. Epub 2011 Oct 27. PMID: 22037950.

47. Shi Q, Chen J, Pongmoragot J, Lanthier S, Saposnik G. Prevalence of Fabry disease in stroke patients--a systematic review and meta-analysis. J Stroke Cerebrovasc Dis. 2014 May-Jun;23(5):985-92. doi:10.1016/j.jstrokecerebrovasdis.2013.08.010. Epub 2013 Oct 11. PMID: 24126289.

48. Grewal RP. Stroke in Fabry's disease. J Neurol. 1994 Jan;241(3):153-6. doi: 10.1007/BF00868342. PMID: 8164017.

49. Grewal RP. Psychiatric disorders in patients with Fabry's disease. Int J Psychiatry Med. 1993;23(3):307-12. doi: 10.2190/JKFW-3WXK-QA7N-BYLN. PMID:8270359.

50. Linthorst GE, Ginsberg L. Prevalence of Fabry disease in TIA/stroke cohorts. What defines Fabry disease? Eur J Neurol. 2012 Nov;19(11):1383-4. doi: 10.1111/j.1468-1331.2012.03763.x. Epub 2012 Jun 4. PMID: 22672596.

51. Dubuc V, Moore DF, Gioia LC, Saposnik G, Selchen D, Lanthier S. Prevalence of Fabry disease in young patients with cryptogenic ischemic stroke. J Stroke Cerebrovasc Dis. 2013 Nov;22(8):1288-92. doi:10.1016/j.jstrokecerebrovasdis.2012.10.005. Epub 2012 Nov 17. PMID: 23168217.

52. Masson C, Cissé I, Simon V, Insalaco P, Audran M. Fabry disease: a review. Joint Bone Spine. 2004 Sep;71(5):381-3. doi: 10.1016/j.jbspin.2003.10.015. PMID:15474388.

53. Zhao Y, Zhu Y, Li F, Sun Y, Ma W, Wu Y, Zhang W, Wang Z, Yuan Y, Huang Y. Brain MRI correlations with disease burden and biomarkers in Fabry disease. J Neurol. 2023 Oct;270(10):4939-4948. doi: 10.1007/s00415-023-11826-8. Epub 2023 Jun 25. PMID: 37356023; PMCID: PMC10511580.

54. Liu D, Hu K, Schmidt M, Müntze J, Maniuc O, Gensler D, Oder D, Salinger T, Weidemann F, Ertl G, Frantz S, Wanner C, Nordbeck P. Value of the CHA₂DS₂-VASc score and Fabry-specific score for predicting new-onset or recurrent stroke/TIA in Fabry disease patients without atrial fibrillation. Clin Res Cardiol. 2018 Dec;107(12):1111-1121. doi: 10.1007/s00392-018-1285-4. Epub 2018 May 24. PMID: 29797054; PMCID: PMC6244978.

55. Saarinen JT, Sillanpää N, Kantola I. A male Fabry disease patient treated with intravenous thrombolysis for acute ischemic stroke. J Clin Neurosci. 2015 Feb;22(2):423-5. doi: 10.1016/j.jocn.2014.07.021. Epub 2014 Oct 28. PMID: 25439755.

56. Mehta A, Beck M, Eyskens F, Feliciani C, Kantola I, Ramaswami U, Rolfs A, Rivera A, Waldek S, Germain DP. Fabry disease: a review of current management strategies. QJM. 2010 Sep;103(9):641-59. doi: 10.1093/qjmed/hcq117. Epub 2010 Jul 21. PMID: 20660166.

57. Møller AT, Jensen TS. Neurological manifestations in Fabry's disease. Nat Clin Pract Neurol. 2007 Feb;3(2):95-106. doi: 10.1038/ncpneuro0407. PMID: 17279083.

58. Romani I, Nencini P, Sarti C, Pracucci G, Zedde M, Nucera A, Cianci V, Moller J, Toni D, Orsucci D, Casella C, Pinto V, Palumbo P, Barbarini L, Bella R, Ragno M, Scoditti U, Mezzapesa DM, Tassi R, Diomedi M, Cavallini A, Volpi G, Chiti A, Bigliardi G, Sacco S, Linoli G, Ricci S, Giordano A, Bonetti B, Rasura M, Cecconi E, Princiotta Cariddi L, Currò Dossi R, Melis M, Consoli D, Guidetti D, Biagini S, Accavone D, Inzitari D. Fabry-Stroke Italian Registry (FSIR): a nationwide, prospective, observational study about incidence and characteristics of Fabry-related stroke in young-adults. Presentation of the study protocol. Neurol Sci. 2022 Apr;43(4):2433-2439. doi: 10.1007/s10072-021-05615-2. Epub 2021 Oct 5. PMID: 34609660; PMCID: PMC8918192.

59. Kilarski LL, Rutten-Jacobs LC, Bevan S, Baker R, Hassan A, Hughes DA, Markus HS; UK Young Lacunar Stroke DNA Study. Prevalence of CADASIL and Fabry Disease in a Cohort of MRI Defined Younger Onset Lacunar Stroke. PLoS One. 2015 Aug 25;10(8):e0136352. doi: 10.1371/journal.pone.0136352. PMID: 26305465; PMCID:PMC4549151.

60. Germain DP. Fabry disease: recent advances in enzyme replacement therapy. Expert Opin Investig Drugs. 2002 Oct;11(10):1467-76. doi: 10.1517/13543784.11.10.1467. PMID: 12387706.

61. Siniscalchi A, La Russa A, Lochner P, Petrone A, Russo B. Cerebral Vasoreactivity in a Fabry Disease Patient. Curr Med Imaging. 2022;18(13):1443-1446. doi: 10.2174/1573405618666220518101844. PMID: 35593334.

62. Thijs V, Grittner U, Fazekas F, McCabe DJH, Giese AK, Kessler C, Martus P, Norrving B, Ringelstein EB, Schmidt R, Tanislav C, Putaala J, Tatlisumak T, von Sarnowski B, Rolfs A, Enzinger C; Stroke in Fabry (SIFAP1) Investigators. Dolichoectasia and Small Vessel Disease in Young Patients With Transient Ischemic Attack and Stroke. Stroke. 2017 Sep;48(9):2361-2367. doi: 10.1161/STROKEAHA.117.017406. Epub 2017 Jul 28. PMID: 28754833.

63. Winter Y, Hilz M, Beuschlein F, Tsukimura T, Seifritz E, Lenders M, Brand E, Hennermann JB, Nowak A. Screening for health-related quality of life and its determinants in Fabry disease: A cross-sectional multicenter study. Mol Genet Metab. 2023 Nov;140(3):107692. doi: 10.1016/j.ymgme.2023.107692. Epub 2023 Aug 29. PMID: 37703724.

64. Müller MJ, Fellgiebel A, Scheurich A, Whybra C, Beck M, Müller KM. Recurrent brief depression in a female patient with Fabry disease. Bipolar Disord. 2006 Aug;8(4):418-9. doi: 10.1111/j.1399-5618.2006.00362.x. PMID: 16879144.

65. Laney DA, Gruskin DJ, Fernhoff PM, Cubells JF, Ousley OY, Hipp H, Mehta AJ. Social-adaptive and psychological functioning of patients affected by Fabry disease. J Inherit Metab

Dis. 2010 Dec;33 Suppl 3:S73-81. doi: 10.1007/s10545-009-9025-6. Epub 2010 Jan 20. PMID: 20087663.

66. Sadek J, Shellhaas R, Camfield CS, Camfield PR, Burley J. Psychiatric findings in four female carriers of Fabry disease. Psychiatr Genet. 2004 Dec;14(4):199-201. doi: 10.1097/00041444-200412000-00006. PMID: 15564893.

67. Laaksonen SM, Röyttä M, Jääskeläinen SK, Kantola I, Penttinen M, Falck B. Neuropathic symptoms and findings in women with Fabry disease. Clin Neurophysiol. 2008 Jun;119(6):1365-72. doi: 10.1016/j.clinph.2008.02.004. Epub 2008 Apr 1. PMID: 18387337.

68. Mancuso M, Arnold M, Bersano A, Burlina A, Chabriat H, Debette S, Enzinger C, Federico A, Filla A, Finsterer J, Hunt D, Lesnik Oberstein S, Tournier-Lasserve E, Markus HS. Monogenic cerebral small-vessel diseases: diagnosis and therapy. Consensus recommendations of the European Academy of Neurology. Eur J Neurol. 2020 Jun;27(6):909-927. doi: 10.1111/ene.14183. Epub 2020 Mar 20. PMID: 32196841.

NEUROIMAGING

A comprehensive literature search was conducted to identify relevant studies on neuroradiological findings in Fabry disease. The search strategy utilized Medical Subject Headings (MeSH) and keyword terms to ensure a broad yet targeted selection of studies. The search was structured into two main components: (1) Fabry disease and its association with cerebrovascular abnormalities and (2) neuroimaging modalities specific to the central nervous system.

Fabry disease was searched using MeSH terms and synonyms, including "Fabry Disease" and "Anderson-Fabry Disease." The second component included neuroimaging-related terms such as "Brain Magnetic Resonance Imaging," "Computed Tomography of the Brain," "Transcranial Doppler Ultrasonography," "Brain Positron Emission Tomography," and "Brain Single-Photon Emission Computed Tomography." These terms were combined using Boolean operators (*AND*, *OR*) to refine the search strategy and retrieve relevant studies.

The search was conducted in major biomedical databases, ensuring comprehensive coverage of peer-reviewed studies relevant to the topic. The outcomes of this Delphi process will provide guidance for standardized neuroimaging evaluation in Fabry disease, ultimately improving early diagnosis, monitoring strategies, and treatment decisions.

PRISMA

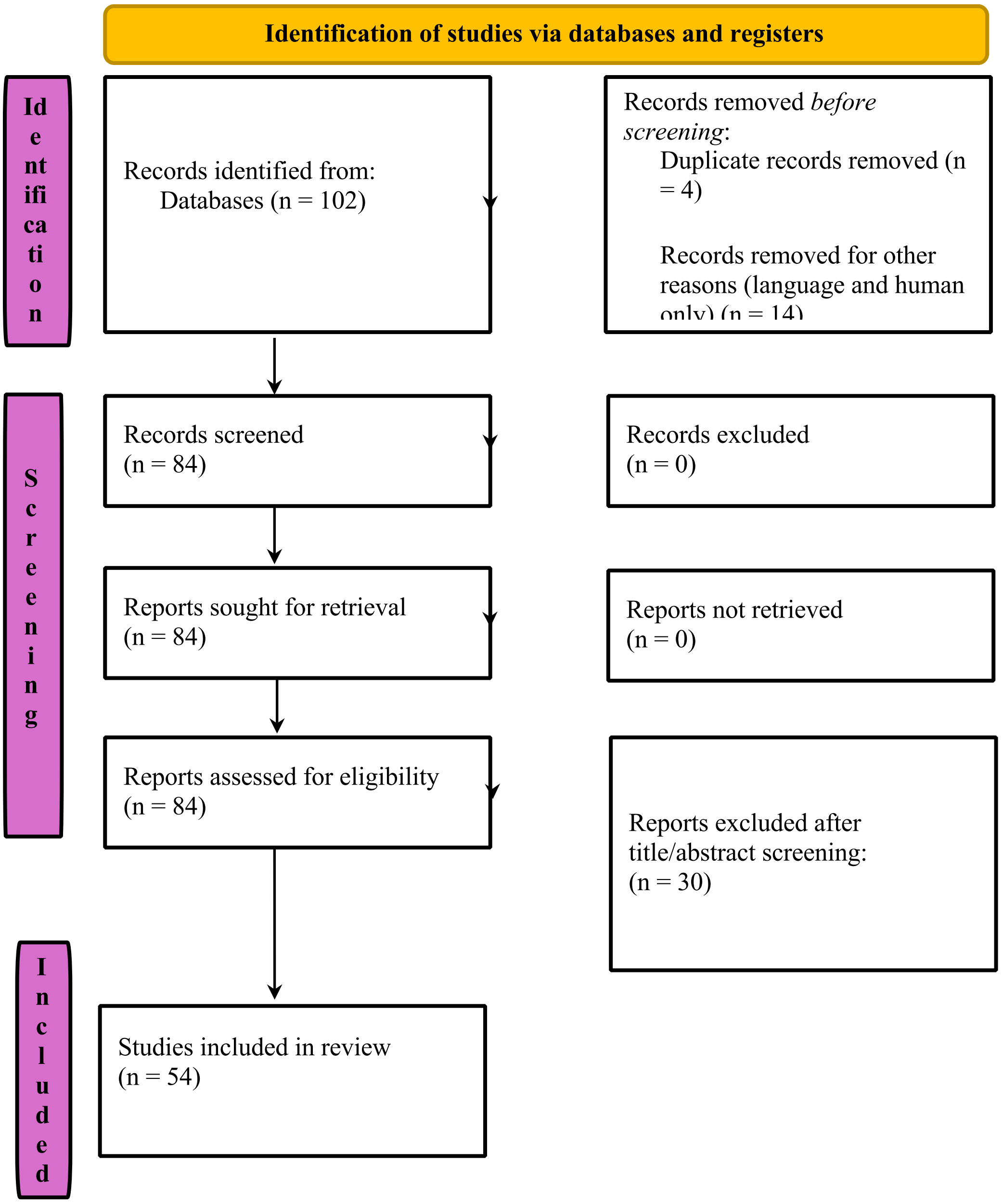

REFERENCES

1: Korsholm K, Feldt-Rasmussen U, Granqvist H, Højgaard L, Bollinger B, Rasmussen AK, Law I. Positron Emission Tomography and Magnetic Resonance Imaging of the Brain in Fabry Disease: A Nationwide, Long-Time, Prospective Follow-Up. PLoS One. 2015 Dec 2;10(12):e0143940. doi: 10.1371/journal.pone.0143940. PMID: 26629990; PMCID: PMC4667906.

2: Lee D, Kim JS, Kim DR, Ihm CG, Yoon SS. Fabry disease with lenticular

degeneration without pulvinar sign. Neurol Sci. 2017 May;38(5):913-914. doi: 10.1007/s10072-017-2819-7. Epub 2017 Jan 19. PMID: 28105577.

3: Körver S, Vergouwe M, Hollak CEM, van Schaik IN, Langeveld M. Development and clinical consequences of white matter lesions in Fabry disease: a systematic review. Mol Genet Metab. 2018 Nov;125(3):205-216. doi: 10.1016/j.ymgme.2018.08.014. Epub 2018 Sep 5. PMID: 30213639.

4: Farooq S, Hiner BC, Rhead WJ, Kirschner AL, Chelimsky TC. Characteristic Pulvinar Sign in Pseudo-α-galactosidase Deficiency Syndrome. JAMA Neurol. 2016 Aug 1;73(8):1020-1. doi: 10.1001/jamaneurol.2016.0268. Erratum in: JAMA Neurol. 2016 Aug 1;73(8):1033. doi: 10.1001/jamaneurol.2016.3034. PMID: 27323002.

5: Menzies DG, Campbell IW, Kean DM. Magnetic resonance imaging in Fabry's disease. J Neurol Neurosurg Psychiatry. 1988 Sep;51(9):1240-1. doi: 10.1136/jnnp.51.9.1240. PMID: 3147319; PMCID: PMC1033041.

6: Gago MF, Azevedo O, Guimarães A, Teresa Vide A, Lamas NJ, Oliveira TG, Gaspar P, Bicho E, Miltenberger-Miltenyi G, Ferreira J, Sousa N. Parkinson's Disease and Fabry Disease: Clinical, Biochemical and Neuroimaging Analysis of Three Pedigrees. J Parkinsons Dis. 2020;10(1):141-152. doi: 10.3233/JPD-191704. PMID: 31594250; PMCID: PMC7029331.

7: Körver S, Geurtsen GJ, Hollak CEM, van Schaik IN, Longo MGF, Lima MR, Dijkgraaf MGW, Langeveld M. Cognitive functioning and depressive symptoms in Fabry disease: A follow-up study. J Inherit Metab Dis. 2020 Sep;43(5):1070-1081. doi: 10.1002/jimd.12271. Epub 2020 Jun 25. PMID: 32510623; PMCID: PMC7540266.

8: Lyndon D, Davagnanam I, Wilson D, Jichi F, Merwick A, Bolsover F, Jager HR, Cipolotti L, Wheeler-Kingshott C, Hughes D, Murphy E, Lachmann R, Werring DJ. MRI-visible perivascular spaces as an imaging biomarker in Fabry disease. J Neurol. 2021 Mar;268(3):872-878. doi: 10.1007/s00415-020-10209-7. Epub 2020 Oct 19. PMID: 33078310; PMCID: PMC7914182.

9: Yagita Y, Sakai N, Miwa K, Ohara N, Tanaka M, Sakaguchi M, Kitagawa K, Mochizuki H. Magnetic Resonance Imaging Findings Related to Stroke Risk in Japanese Patients With Fabry Disease. Stroke. 2019 Sep;50(9):2571-2573. doi:

10.1161/STROKEAHA.119.025528. Epub 2019 Jul 24. PMID: 31337300.

10: Pontillo G, Tranfa M, Scaravilli A, Monti S, Capuano I, Riccio E, Rizzo M, Brunetti A, Palma G, Pisani A, Cocozza S. In vivo demonstration of globotriaosylceramide brain accumulation in Fabry Disease using MR Relaxometry. Neuroradiology. 2024 Sep;66(9):1593-1601. doi: 10.1007/s00234-024-03380-5. Epub 2024 May 21. PMID: 38771548; PMCID: PMC11322198.

11: Russo C, Pontillo G, Pisani A, Saccà F, Riccio E, Macera A, Rusconi G, Stanzione A, Borrelli P, Brescia Morra V, Tedeschi E, Brunetti A, Cocozza S, Palma G. Striatonigral involvement in Fabry Disease: A quantitative and volumetric Magnetic Resonance Imaging study. Parkinsonism Relat Disord. 2018 Dec;57:27-32. doi: 10.1016/j.parkreldis.2018.07.011. Epub 2018 Jul 21. PMID: 30054181.

12: Paavilainen T, Lepomäki V, Saunavaara J, Borra R, Nuutila P, Kantola I, Parkkola R. Diffusion tensor imaging and brain volumetry in Fabry disease patients. Neuroradiology. 2013 May;55(5):551-8. doi: 10.1007/s00234-012-1131-8. Epub 2013 Jan 5. PMID: 23292181.

13: Ulivi L, Kanber B, Prados F, Davagnanam I, Merwick A, Chan E, Williams F, Hughes D, Murphy E, Lachmann RH, Wheeler-Kingshott CAMG, Cipolotti L, Werring DJ. White matter integrity correlates with cognition and disease severity in Fabry disease. Brain. 2020 Dec 5;143(11):3331-3342. doi: 10.1093/brain/awaa282. PMID: 33141169.

14: Manara R, Carlier RY, Righetto S, Citton V, Locatelli G, Colas F, Ermani M, Germain DP, Burlina A. Basilar Artery Changes in Fabry Disease. AJNR Am J Neuroradiol. 2017 Mar;38(3):531-536. doi: 10.3174/ajnr.A5069. Epub 2017 Jan 26. PMID: 28126747; PMCID: PMC7960013.

15: Schermuly I, Müller MJ, Müller KM, Albrecht J, Keller I, Yakushev I, Beck M, Fellgiebel A. Neuropsychiatric symptoms and brain structural alterations in Fabry disease. Eur J Neurol. 2011 Feb;18(2):347-353. doi: 10.1111/j.1468-1331.2010.03155.x. PMID: 20636371.

16: Körver S, Geurtsen GJ, Hollak CEM, van Schaik IN, Longo MGF, Lima MR,

Vedolin L, Dijkgraaf MGW, Langeveld M. Depressive symptoms in Fabry disease: the importance of coping, subjective health perception and pain. Orphanet J Rare Dis. 2020 Jan 28;15(1):28. doi: 10.1186/s13023-020-1307-y. PMID: 31992347; PMCID: PMC6986064.

17: Lelieveld IM, Böttcher A, Hennermann JB, Beck M, Fellgiebel A. Eight-Year Follow-Up of Neuropsychiatric Symptoms and Brain Structural Changes in Fabry Disease. PLoS One. 2015 Sep 4;10(9):e0137603. doi: 10.1371/journal.pone.0137603. PMID: 26340726; PMCID: PMC4560446.

18: Reisin RC, Romero C, Marchesoni C, Nápoli G, Kisinovsky I, Cáceres G, Sevlever G. Brain MRI findings in patients with Fabry disease. J Neurol Sci. 2011 Jun 15;305(1-2):41-4. doi: 10.1016/j.jns.2011.03.020. Epub 2011 Apr 3. PMID: 21463870.

19: Cocozza S, Russo C, Pisani A, Olivo G, Riccio E, Cervo A, Pontillo G, Feriozzi S, Veroux M, Battaglia Y, Concolino D, Pieruzzi F, Mignani R, Borrelli P, Imbriaco M, Brunetti A, Tedeschi E, Palma G. Redefining the Pulvinar Sign in Fabry Disease. AJNR Am J Neuroradiol. 2017 Dec;38(12):2264-2269. doi: 10.3174/ajnr.A5420. Epub 2017 Oct 19. PMID: 29051208; PMCID: PMC7963734.

20: Azevedo E, Mendes A, Seixas D, Santos R, Castro P, Ayres-Basto M, Rosengarten B, Oliveira JP. Functional transcranial Doppler: presymptomatic changes in Fabry disease. Eur Neurol. 2012;67(6):331-7. doi: 10.1159/000337906. Epub 2012 May 4. PMID: 22572628.

21: Körver S, Longo MGF, Lima MR, Hollak CEM, El Sayed M, van Schaik IN, Vedolin L, Dijkgraaf MGW, Langeveld M. Determinants of cerebral radiological progression in Fabry disease. J Neurol Neurosurg Psychiatry. 2020 Jul;91(7):756-763. doi: 10.1136/jnnp-2019-322268. Epub 2020 Apr 21. PMID: 32317398.

22: Gregoire SM, Brown MM, Collas DM, Jacob P, Lachmann RH, Werring DJ. Posterior circulation strokes without systemic involvement as the presenting feature of Fabry disease. J Neurol Neurosurg Psychiatry. 2009 Dec;80(12):1414-6. doi: 10.1136/jnnp.2008.158790. PMID: 19917829.

23: Stefaniak JD, Parkes LM, Parry-Jones AR, Potter GM, Vail A, Jovanovic A,

Smith CJ. Enzyme replacement therapy and white matter hyperintensity progression in Fabry disease. Neurology. 2018 Oct 9;91(15):e1413-e1422. doi: 10.1212/WNL.0000000000006316. Epub 2018 Sep 12. PMID: 30209238; PMCID: PMC6177273.

24: Marino S, Borsini W, Buchner S, Mortilla M, Stromillo ML, Battaglini M, Giorgio A, Bramanti P, Federico A, De Stefano N. Diffuse structural and metabolic brain changes in Fabry disease. J Neurol. 2006 Apr;253(4):434-40. doi: 10.1007/s00415-005-0020-z. Epub 2006 Mar 20. PMID: 16541218.

25: Cocozza S, Schiavi S, Pontillo G, Battocchio M, Riccio E, Caccavallo S, Russo C, Di Risi T, Pisani A, Daducci A, Brunetti A. Microstructural damage of the cortico-striatal and thalamo-cortical fibers in Fabry disease: a diffusion MRI tractometry study. Neuroradiology. 2020 Nov;62(11):1459-1466. doi: 10.1007/s00234-020-02497-7. Epub 2020 Jul 22. PMID: 32700105; PMCID: PMC7568710.

26: Yamadera M, Yokoe M, Beck G, Mihara M, Oe H, Yamamoto Y, Sakoda S. Amelioration of white-matter lesions in a patient with Fabry disease. J Neurol Sci. 2009 Apr 15;279(1-2):118-20. doi: 10.1016/j.jns.2008.12.028. Epub 2009 Jan 29. PMID: 19185318.

27: Burlina AP, Manara R, Caillaud C, Laissy JP, Severino M, Klein I, Burlina A, Lidove O. The pulvinar sign: frequency and clinical correlations in Fabry disease. J Neurol. 2008 May;255(5):738-44. doi: 10.1007/s00415-008-0786-x. Epub 2008 Feb 26. PMID: 18297328.

28: Baas KPA, Everard AJ, Körver S, van Dussen L, Coolen BF, Strijkers GJ, Hollak CEM, Nederveen AJ. Progressive Changes in Cerebral Apparent Diffusion Values in Fabry Disease: A 5-Year Follow-up MRI Study. AJNR Am J Neuroradiol. 2023 Oct;44(10):1157-1164. doi: 10.3174/ajnr.A8001. PMID: 37770205; PMCID: PMC10549936.

29: Fellgiebel A, Mazanek M, Whybra C, Beck M, Hartung R, Müller KM, Scheurich A, Dellani PR, Stoeter P, Müller MJ. Pattern of microstructural brain tissue alterations in Fabry disease: a diffusion-tensor imaging study. J Neurol. 2006 Jun;253(6):780-7. doi: 10.1007/s00415-006-0118-y. Epub 2006 Mar 6. PMID: 16511647.

30: Moore DF, Altarescu G, Barker WC, Patronas NJ, Herscovitch P, Schiffmann R. White matter lesions in Fabry disease occur in 'prior' selectively hypometabolic and hyperperfused brain regions. Brain Res Bull. 2003 Dec 30;62(3):231-40. doi: 10.1016/j.brainresbull.2003.09.021. PMID: 14698356.

31: Cocozza S, Pontillo G, Quarantelli M, Saccà F, Riccio E, Costabile T, Olivo G, Brescia Morra V, Pisani A, Brunetti A, Tedeschi E; AFFINITY study group. Default mode network modifications in Fabry disease: A resting-state fMRI study with structural correlations. Hum Brain Mapp. 2018 Apr;39(4):1755-1764. doi: 10.1002/hbm.23949. Epub 2018 Jan 9. PMID: 29315984; PMCID: PMC6866450.

32: Jardim LB, Aesse F, Vedolin LM, Pitta-Pinheiro C, Marconato J, Burin MG, Cecchin C, Netto CB, Matte US, Pereira F, Kalakun L, Giugliani R. White matter lesions in Fabry disease before and after enzyme replacement therapy: a 2-year follow-up. Arq Neuropsiquiatr. 2006 Sep;64(3B):711-7. doi: 10.1590/s0004-282x2006000500002. PMID: 17057872.

33: Fellgiebel A, Keller I, Marin D, Müller MJ, Schermuly I, Yakushev I, Albrecht J, Bellhäuser H, Kinateder M, Beck M, Stoeter P. Diagnostic utility of different MRI and MR angiography measures in Fabry disease. Neurology. 2009 Jan 6;72(1):63-8. doi: 10.1212/01.wnl.0000338566.54190.8a. PMID: 19122032.

34: Gavazzi C, Borsini W, Guerrini L, Della Nave R, Rocca MA, Tessa C, Buchner S, Belli G, Filippi M, Villari N, Mascalchi M. Subcortical damage and cortical functional changes in men and women with Fabry disease: a multifaceted MR study. Radiology. 2006 Nov;241(2):492-500. doi: 10.1148/radiol.2412051122. PMID: 17057070.

35: Underhill HR, Golden-Grant K, Garrett LT, Uhrich S, Zielinski BA, Scott CR. Detecting the effects of Fabry disease in the adult human brain with diffusion tensor imaging and fast bound-pool fraction imaging. J Magn Reson Imaging. 2015 Dec;42(6):1611-22. doi: 10.1002/jmri.24952. Epub 2015 May 27. PMID: 26018987; PMCID: PMC4662657.

36: Buechner S, Moretti M, Burlina AP, Cei G, Manara R, Ricci R, Mignani R, Parini R, Di Vito R, Giordano GP, Simonelli P, Siciliano G, Borsini W. Central

nervous system involvement in Anderson-Fabry disease: a clinical and MRI retrospective study. J Neurol Neurosurg Psychiatry. 2008 Nov;79(11):1249-54. doi: 10.1136/jnnp.2008.143693. Epub 2008 Jun 5. PMID: 18535022.

37: Phyu P, Merwick A, Davagnanam I, Bolsover F, Jichi F, Wheeler-Kingshott C, Golay X, Hughes D, Cipolotti L, Murphy E, Lachmann RH, Werring DJ. Increased resting cerebral blood flow in adult Fabry disease: MRI arterial spin labeling study. Neurology. 2018 Apr 17;90(16):e1379-e1385. doi: 10.1212/WNL.0000000000005330. Epub 2018 Mar 21. PMID: 29661900; PMCID: PMC5902785.

38: Tedeschi G, Bonavita S, Banerjee TK, Virta A, Schiffmann R. Diffuse central neuronal involvement in Fabry disease: a proton MRS imaging study. Neurology. 1999 May 12;52(8):1663-7. doi: 10.1212/wnl.52.8.1663. PMID: 10331696.

39: Albrecht J, Dellani PR, Müller MJ, Schermuly I, Beck M, Stoeter P, Gerhard A, Fellgiebel A. Voxel based analyses of diffusion tensor imaging in Fabry disease. J Neurol Neurosurg Psychiatry. 2007 Sep;78(9):964-9. doi: 10.1136/jnnp.2006.112987. Epub 2007 Apr 20. PMID: 17449543; PMCID: PMC2117852.

40: Fellgiebel A, Gartenschläger M, Wildberger K, Scheurich A, Desnick RJ, Sims K. Enzyme replacement therapy stabilized white matter lesion progression in Fabry disease. Cerebrovasc Dis. 2014;38(6):448-56. doi: 10.1159/000369293. Epub 2014 Dec 11. PMID: 25502511.

41: Ugga L, Cocozza S, Pontillo G, Russo C, Brescia Morra V, Lanzillo R, Riccio E, Pisani A, Brunetti A. Absence of infratentorial lesions in Fabry disease contributes to differential diagnosis with multiple sclerosis. Brain Behav. 2018 Nov;8(11):e01121. doi: 10.1002/brb3.1121. Epub 2018 Oct 2. PMID: 30277321; PMCID: PMC6236241.

42: Moore DF, Ye F, Schiffmann R, Butman JA. Increased signal intensity in the pulvinar on T1-weighted images: a pathognomonic MR imaging sign of Fabry disease. AJNR Am J Neuroradiol. 2003 Jun-Jul;24(6):1096-101. PMID: 12812932; PMCID: PMC8149001.

43: Rost NS, Cloonan L, Kanakis AS, Fitzpatrick KM, Azzariti DR, Clarke V,

Lourenco CM, Germain DP, Politei JM, Homola GA, Sommer C, Üçeyler N, Sims KB. Determinants of white matter hyperintensity burden in patients with Fabry disease. Neurology. 2016 May 17;86(20):1880-6. doi: 10.1212/WNL.0000000000002673. Epub 2016 Apr 20. PMID: 27164662; PMCID: PMC4873685.

44: Miwa K, Yagita Y, Sakaguchi M, Kitagawa K, Sakai N, Mochizuki H. Effect of Enzyme Replacement Therapy on Basilar Artery Diameter in Male Patients With Fabry Disease. Stroke. 2019 Apr;50(4):1010-1012. doi: 10.1161/STROKEAHA.118.024426. PMID: 30852964.

45: Cocozza S, Olivo G, Riccio E, Russo C, Pontillo G, Ugga L, Migliaccio S, de Rosa D, Feriozzi S, Veroux M, Battaglia Y, Concolino D, Pieruzzi F, Tuttolomondo A, Caronia A, Russo CV, Lanzillo R, Brescia Morra V, Imbriaco M, Brunetti A, Tedeschi E, Pisani A. Corpus callosum involvement: a useful clue for differentiating Fabry Disease from Multiple Sclerosis. Neuroradiology. 2017 Jun;59(6):563-570. doi: 10.1007/s00234-017-1829-8. Epub 2017 Apr 6. PMID: 28386689.

46: Takanashi J, Barkovich AJ, Dillon WP, Sherr EH, Hart KA, Packman S. T1 hyperintensity in the pulvinar: key imaging feature for diagnosis of Fabry disease. AJNR Am J Neuroradiol. 2003 May;24(5):916-21. PMID: 12748094; PMCID: PMC7975809.

47: Moore DF, Ye F, Brennan ML, Gupta S, Barshop BA, Steiner RD, Rhead WJ, Brady RO, Hazen SL, Schiffmann R. Ascorbate decreases Fabry cerebral hyperperfusion suggesting a reactive oxygen species abnormality: an arterial spin tagging study. J Magn Reson Imaging. 2004 Oct;20(4):674-83. doi: 10.1002/jmri.20162. PMID: 15390234.

48: Cocozza S, Pisani A, Olivo G, Saccà F, Ugga L, Riccio E, Migliaccio S, Brescia Morra V, Brunetti A, Quarantelli M, Tedeschi E. Alterations of functional connectivity of the motor cortex in Fabry disease: An RS-fMRI study. Neurology. 2017 May 9;88(19):1822-1829. doi: 10.1212/WNL.0000000000003913. Epub 2017 Apr 12. PMID: 28404798.

49: Moore DF, Schiffmann R, Ulug AM. Elevated CNS average diffusion constant in

Fabry disease. Acta Paediatr Suppl. 2002;91(439):67-8. doi: 10.1111/j.1651-2227.2002.tb03114.x. PMID: 12572846.

50: Crutchfield KE, Patronas NJ, Dambrosia JM, Frei KP, Banerjee TK, Barton NW, Schiffmann R. Quantitative analysis of cerebral vasculopathy in patients with Fabry disease. Neurology. 1998 Jun;50(6):1746-9. doi: 10.1212/wnl.50.6.1746. PMID: 9633721.

51: Grewal RP, McLatchey SK. Cerebrovascular manifestations in a female carrier of Fabry's disease. Acta Neurol Belg. 1992;92(1):36-40. PMID: 1546524.

52: Wardlaw, J. M., Smith, E. E., Biessels, G. J., Cordonnier, C., Fazekas, F., Frayne, R., ... & Dichgans, M. (2013). "Neuroimaging standards for research into small vessel disease and its contribution to ageing and neurodegeneration." *The Lancet Neurology, 12*(8), 822-838.

53: Cocozza S, Russo C, Pontillo G, Pisani A, Brunetti A. Neuroimaging in Fabry disease: current knowledge and future directions. Insights Imaging. 2018 Dec;9(6):1077-1088. doi: 10.1007/s13244-018-0664-8. Epub 2018 Nov 2. PMID: 30390274; PMCID: PMC6269338.

54: Scaravilli A, Capasso S, Ugga L, Capuano I, Risi TD, Pontillo G, Riccio E, Tranfa M, Pisani A, Brunetti A, Cocozza S. Clinical and pathophysiological correlates of basilar artery measurements in Fabry Disease. AJNR Am J Neuroradiol. 2024 Jul 12:ajnr.A8403. doi: 10.3174/ajnr.A8403. Epub ahead of print. PMID: 38997124.

PERIPHERAL NERVOUS SYSTEM INVOLVEMENT

VEGETATIVE INVOLVEMENT

A comprehensive literature search was conducted to identify relevant studies on Peripheral Nervous System involvement and gastrointestinal dysmotility in Fabry disease. The search strategy utilized Medical Subject Headings (MeSH) and keyword terms to ensure a broad yet targeted selection of studies.

Fabry disease was searched using MeSH terms and synonyms, including "Fabry Disease"[MeSH], Anderson-Fabry Disease, and alpha-galactosidase A deficiency. Peripheral nervous system involvement was captured using terms such as "Peripheral Nervous System Diseases"[MeSH], "Peripheral Neuropathy", "Neuralgia"[MeSH], and "Neuropathic Pain"[MeSH]. Gastrointestinal symptoms and motility disorders were explored using "Gastrointestinal Diseases"[MeSH], "Gastrointestinal Motility"[MeSH], "Abdominal Pain"[MeSH], "Diarrhea"[MeSH], and "Constipation"[MeSH]. To assess patient-centered outcomes, the search included "Patient Reported Outcome Measures"[MeSH], "Quality of Life"[MeSH], "Self Report"[MeSH], and "Questionnaires"[MeSH].

The search was conducted in major biomedical databases, ensuring comprehensive coverage of peer-reviewed studies relevant to the topic. The outcomes of this Delphi process will provide guidance for standardized evaluation in Fabry disease with regards to peripheral neuropathy, gastrointestinal disturbance and their relative PROMs, ultimately improving early diagnosis, monitoring strategies, and treatment decisions.

PRISMA

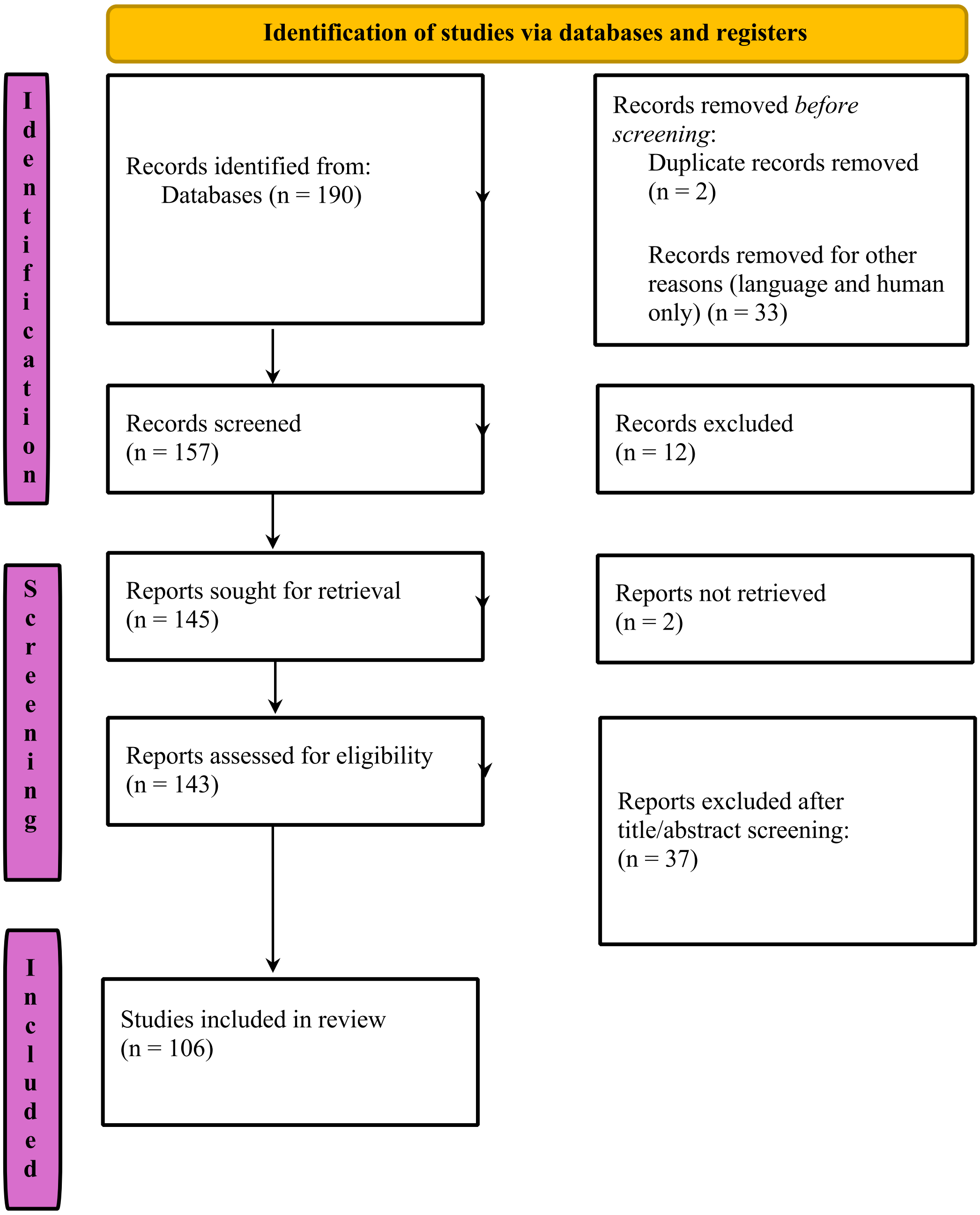

REFERENCES

1. Pain in Fabry Disease: Practical Recommendations for Diagnosis and Treatment. Politei JM, Bouhassira D, Germain DP, Goizet C, Guerrero-Sola A, Hilz MJ, Hutton EJ, Karaa A, Liguori R, Üçeyler N, Zeltzer LK, Burlina A.CNS Neurosci Ther. 2016 Jul;22(7):568-76
2. Non-specific gastrointestinal features: Could it be Fabry disease? Hilz MJ, Arbustini E, Dagna L, Gasbarrini A, Goizet C, Lacombe D, Liguori R, Manna R, Politei J, Spada M, Burlina A.Dig Liver Dis. 2018 May;50(5):429-437
3. Early diagnosis of peripheral nervous system involvement in Fabry disease and treatment of neuropathic pain: the report of an expert panel. Burlina AP, Sims KB, Politei JM, Bennett GJ, Baron R, Sommer C, Møller AT, Hilz MJ.BMC Neurol. 2011 May 27;11:61.

4. Altered globotriaosylceramide accumulation and mucosal neuronal fiber density in the colon of the Fabry disease mouse model. Masotti M, Delprete C, Dothel G, Donadio V, Rimondini R, Politei JM, Liguori R, Caprini M.Neurogastroenterol Motil. 2019 Mar;31(3):e13529
5. Skin globotriaosylceramide 3 deposits are specific to Fabry disease with classical mutations and associated with small fibre neuropathy. Liguori R, Incensi A, de Pasqua S, Mignani R, Fileccia E, Santostefano M, Biagini E, Rapezzi C, Palmieri S, Romani I, Borsini W, Burlina A, Bombardi R, Caprini M, Avoni P, Donadio V.PLoS One. 2017 Jul 3;12(7):e0180581.
6. Pain related channels are differentially expressed in neuronal and non-neuronal cells of glabrous skin of fabry knockout male mice. Lakomá J, Rimondini R, Donadio V, Liguori R, Caprini M.PLoS One. 2014 Oct 22;9(10):e108641.
7. Small fiber neuropathy in female patients with fabry disease. Liguori R, Di Stasi V, Bugiardini E, Mignani R, Burlina A, Borsini W, Baruzzi A, Montagna P, Donadio V.Muscle Nerve. 2010 Mar;41(3):409-12
8. Increased expression of Trpv1 in peripheral terminals mediates thermal nociception in Fabry disease mouse model. Lakomá J, Rimondini R, Ferrer Montiel A, Donadio V, Liguori R, Caprini M.Mol Pain. 2016 Aug 16;12:1744806916663729.
9. Fabry disease pain: patient and preclinical parallels. Burand AJ Jr, Stucky CL.Pain. 2021 May 1;162(5):1305-1321
10. Review of Mechanisms, Pharmacological Management, Psychosocial Implications, and Holistic Treatment of Pain in Fabry Disease. Rajan JN, Ireland K, Johnson R, Stepien KM.J Clin Med. 2021 Sep 15;10(18):4168.
11. Pathophysiology and assessment of neuropathic pain in Fabry disease. Schiffmann R, Scott LJ.Acta Paediatr Suppl. 2002;91(439):48-52
12. Mechanisms of neuropathic pain and their importance in Fabry disease. Birklein F.Acta Paediatr Suppl. 2002;91(439):34-7
13. Neuropathic pain in Anderson-Fabry disease: pathology and therapeutic options. MacDermot J, MacDermot KD.Eur J Pharmacol. 2001 Oct 19;429(1-3):121-5.
14. Early diagnosis of peripheral nervous system involvement in Fabry disease and treatment of neuropathic pain: the report of an expert panel. Burlina AP, Sims KB, Politei JM, Bennett GJ, Baron R, Sommer C, Møller AT, Hilz MJ.BMC Neurol. 2011 May 27;11:61.
15. Dysregulation of Immune Response Mediators and Pain-Related Ion Channels Is Associated with Pain-like Behavior in the GLA KO Mouse Model of FabryDisease. Spitzel M, Wagner E, Breyer M, Henniger D, Bayin M, Hofmann L, Mauceri D, Sommer C, Üçeyler N.Cells. 2022 May 24;11(11):1730.
16. The correlation of small fiber neuropathy with pain intensity and age in patients with Fabry's disease: A cross sectional study within a large Taiwanese family. Liao MF, Hsu JL, Fung HC, Kuo HC, Chu CC, Chang HS, Lyu RK, Ro LS.Biomed J. 2022 Apr;45(2):406-413
17. Depressive symptoms in Fabry disease: the importance of coping, subjective health perception and pain. Körver S, Geurtsen GJ, Hollak CEM, van Schaik IN, Longo MGF, Lima MR, Vedolin L, Dijkgraaf MGW, Langeveld M.Orphanet J Rare Dis. 2020 Jan 28;15(1):28.
18. Health-related quality of life in Norwegian adults with Fabry disease: Disease severity, pain, fatigue and psychological distress. Pihlstrøm HK, Weedon-Fekjær MS, Bjerkely BL, von der Lippe C, Ørstavik K, Mathisen P, Heimdal K, Jenssen TG, Dahle DO, Solberg OK, Sigurdardottir S.JIMD Rep. 2021 Jul 16;62(1):56-69

19. Management of pain in Fabry disease in the UK clinical setting: consensus findings from an expert Delphi panel. Stepien KM, Broomfield A, Cole D, Deegan PB, Forshaw-Hulme S, Hughes D, Jovanovic A, Morris L, Muir A, Ramaswami U.Orphanet J Rare Dis. 2023 Jul 21;18(1):203.
20. Pain management strategies for neuropathic pain in Fabry disease--a systematic review. Schuller Y, Linthorst GE, Hollak CE, Van Schaik IN, Biegstraaten M.BMC Neurol. 2016 Feb 24;16:25
21. Globotriaosylceramide-induced reduction of K(Ca)1.1 channel activity and activation of the Notch1 signaling pathway in skin fibroblasts of male Fabrypatients with pain. Rickert V, Kramer D, Schubert AL, Sommer C, Wischmeyer E, Üçeyler N.Exp Neurol. 2020 Feb;324:113134
22. Neuropathic pain in a Fabry disease rat model. Miller JJ, Aoki K, Moehring F, Murphy CA, O'Hara CL, Tiemeyer M, Stucky CL, Dahms NM.JCI Insight. 2018 Mar 22;3(6):e99171
23. Phenotype, disease severity and pain are major determinants of quality of life in Fabry disease: results from a large multicenter cohort study. Arends M, Körver S, Hughes DA, Mehta A, Hollak CEM, Biegstraaten M.J Inherit Metab Dis. 2018 Jan;41(1):141-149
24. Fabry disease: a rare cause of neuropathic pain. Biegstraaten M, Linthorst GE, van Schaik IN, Hollak CE.Curr Pain Headache Rep. 2013 Oct;17(10):365
25. A survey of the pain experienced by males and females with Fabry disease. Gibas AL, Klatt R, Johnson J, Clarke JT, Katz J.Pain Res Manag. 2006 Autumn;11(3):185-92
26. Characterization of pain in fabry disease. Üçeyler N, Ganendiran S, Kramer D, Sommer C.Clin J Pain. 2014 Oct;30(10):915-20.
27. Neuropathy and Fabry disease: pathogenesis and enzyme replacement therapy. Schiffmann R.Acta Neurol Belg. 2006 Jun;106(2):61-5.
28. The relation between small nerve fibre function, age, disease severity and pain in Fabry disease. Biegstraaten M, Binder A, Maag R, Hollak CE, Baron R, van Schaik IN.Eur J Pain. 2011 Sep;15(8):822-9.
29. Comprehensive and differential long-term characterization of the alpha-galactosidase A deficient mouse model of Fabry disease focusing on the sensory system and pain development. Üçeyler N, Biko L, Hose D, Hofmann L, Sommer C.Mol Pain. 2016 May 4;12:1744806916646379
30. Measuring patient experiences in Fabry disease: validation of the Fabry-specific Pediatric Health and Pain Questionnaire (FPHPQ). Ramaswami U, Stull DE, Parini R, Pintos-Morell G, Whybra C, Kalkum G, Rohrbach M, Raluy-Callado M, Beck M, Chen WH, Wiklund I; FOS Investigators.Health Qual Life Outcomes. 2012 Sep 20;10:116.
31. A comprehensive Fabry-related pain questionnaire for adult patients. Üçeyler N, Magg B, Thomas P, Wiedmann S, Heuschmann P, Sommer C.Pain. 2014 Nov;155(11):2301-5
32. Lower limb cold exposure induces pain and prolonged small fiber dysfunction in Fabry patients. Hilz MJ, Stemper B, Kolodny EH.Pain. 2000 Feb;84(2-3):361-5.
33. Nature and prevalence of pain in Fabry disease and its response to enzyme replacement therapy--a retrospective analysis from the Fabry Outcome Survey. Hoffmann B, Beck M, Sunder-Plassmann G, Borsini W, Ricci R, Mehta A; FOS European Investigators.Clin J Pain. 2007 Jul-Aug;23(6):535-42.
34. Nociceptive behavior and central neuropeptidergic dysregulations in male and female mice of a Fabry disease animal model. Rullo L, Posa L, Caputi FF, Stamatakos S, Formaggio F, Caprini M, Liguori R, Candeletti S, Romualdi P.Brain Res Bull. 2021 Oct;175:158-167.

35. Small fibre neuropathy in Fabry disease: a human-derived neuronal in vitro disease model and pilot data. Klein T, Grüner J, Breyer M, Schlegel J, Schottmann NM, Hofmann L, Gauss K, Mease R, Erbacher C, Finke L, Klein A, Klug K, Karl-Schöller F, Vignolo B, Reinhard S, Schneider T, Günther K, Fink J, Dudek J, Maack C, Klopocki E, Seibel J, Edenhofer F, Wischmeyer E, Sauer M, Üçeyler N.Brain Commun. 2024 Apr 3;6(2):fcae095.

36. Clinical significance of small nerve fiber involvement in the early diagnosis and treatment of patients with Fabry disease. Kokotis P, Zompola C, Anastasakis A, Damianaki A, Bountziouka C, Mpora M, Papatheodorou S, Tsivgoulis G.J Neurol Sci. 2023 Oct 15;453:120776

37. Self-administered version of the Fabry-associated pain questionnaire for adult patients. Magg B, Riegler C, Wiedmann S, Heuschmann P, Sommer C, Üçeyler N.Orphanet J Rare Dis. 2015 Sep 17;10:113

38. Effects of enzyme replacement therapy on pain and health related quality of life in patients with Fabry disease: data from FOS (Fabry Outcome Survey). Hoffmann B, Garcia de Lorenzo A, Mehta A, Beck M, Widmer U, Ricci R; FOS European Investigators.J Med Genet. 2005 Mar;42(3):247-52

39. Neuropathic pain as a symptom in autonomic neuropathies and other rare diseases : Small fiber neuropathy: its recognition, diagnosis, and treatment. Fischer F, Dohrn MF, Kapfenberger R, Igharo D, Seeber D, de Moya Rubio E, Pitarokoili K, Börsch N, Mücke M, Rolke R, Schulz JB, Maier A.Schmerz. 2024 Feb;38(1):33-40

40. Fabry disease--pain doctors have to find the missing ones. Naleschinski D, Arning K, Baron R.Pain. 2009 Sep;145(1-2):10-1

41. Enzyme replacement therapy in two Japanese siblings with Fabry disease, and its effectiveness on angiokeratoma and neuropathic pain. Furujo M, Kubo T, Kobayashi M, Ohashi T.Mol Genet Metab. 2013 Nov;110(3):405-10

42. Small fiber neuropathy in Fabry disease. Biegstraaten M, Hollak CE, Bakkers M, Faber CG, Aerts JM, van Schaik IN.Mol Genet Metab. 2012 Jun;106(2):135-41

43. Distal extremity pain as a presenting feature of Fabry's disease. Pagnini I, Borsini W, Cecchi F, Sgalambro A, Olivotto I, Frullini A, Cimaz R.Arthritis Care Res (Hoboken). 2011 Mar;63(3):390-5

44. Pain in Anderson-Fabry's disease. Chowdhury MM, Holt PJ.Lancet. 2001 Mar 17;357(9259):887

45. Pain related channels are differentially expressed in neuronal and non-neuronal cells of glabrous skin of fabry knockout male mice. Lakomá J, Rimondini R, Donadio V, Liguori R, Caprini M.PLoS One. 2014 Oct 22;9(10):e108641.

46. Small fibers in Fabry disease: baseline and follow-up data under enzyme replacement therapy. Üçeyler N, He L, Schönfeld D, Kahn AK, Reiners K, Hilz MJ, Breunig F, Sommer C.J Peripher Nerv Syst. 2011 Dec;16(4):304-14

47. Functional and structural nerve fiber findings in heterozygote patients with Fabry disease. Torvin Møller A, Winther Bach F, Feldt-Rasmussen U, Rasmussen A, Hasholt L, Lan H, Sommer C, Kølvraa S, Ballegaard M, Staehelin Jensen T.Pain. 2009 Sep;145(1-2):237-45.

48. Peripheral neuropathy in Anderson-Fabry disease: its physiology, evaluation and treatment. Politei JM, Pagano MA.Rev Neurol. 2004 May 16-31;38(10):979-83.

49. Neuropathy and Fabry's disease. Lacomis D, Roeske-Anderson L, Mathie L.Muscle Nerve. 2005 Jan;31(1):102-7.

50. Neuropathic symptoms and findings in women with Fabry disease. Laaksonen SM, Röyttä M, Jääskeläinen SK, Kantola I, Penttinen M, Falck B.Clin Neurophysiol. 2008 Jun;119(6):1365-72.

51. Stratification of patients with unclassified pain in the FabryScan database. Forstenpointner J, Moeller P, Sendel M, Reimer M, Hüllemann P, Baron R.J Pain Res. 2019 Jul 23;12:2223-2230

52. Fabry disease: impaired autonomic function. Cable WJ, Kolodny EH, Adams RD.Neurology. 1982 May;32(5):498-502

53. Sensory-specific peripheral nerve pathology in a rat model of Fabry disease. Waltz TB, Burand AJ Jr, Sadler KE, Stucky CL.Neurobiol Pain. 2021 Sep 2;10:100074

54. Dyshidrosis is associated with reduced amplitudes in electrically evoked pain-related potentials in women with Fabry disease. Siedler G, Káhn AK, Weidemann F, Wanner C, Sommer C, Üçeyler N.Clin Neurophysiol. 2019 Apr;130(4):528-536

55. Enzyme replacement therapy improves function of C-, Adelta-, and Abeta-nerve fibers in Fabry neuropathy. Hilz MJ, Brys M, Marthol H, Stemper B, Dütsch M.Neurology. 2004 Apr 13;62(7):1066-72

56. Characterization of small fiber pathology in a mouse model of Fabry disease. Hofmann L, Hose D, Grießhammer A, Blum R, Döring F, Dib-Hajj S, Waxman S, Sommer C, Wischmeyer E, Üçeyler N.Elife. 2018 Oct 17;7:e39300.

57. Enzyme replacement therapy improves peripheral nerve and sweat function in Fabry disease. Schiffmann R, Floeter MK, Dambrosia JM, Gupta S, Moore DF, Sharabi Y, Khurana RK, Brady RO.Muscle Nerve. 2003 Dec;28(6):703-1

58. Morphological and biochemical changes in muscle and peripheral nerve in Fabry's disease. Pellissier JF, Van Hoof F, Bourdet-Bonerandi D, Monier-Faugere MC, Toga M.Muscle Nerve. 1981 Sep-Oct;4(5):381-7.

59. Use of gabapentin to reduce chronic neuropathic pain in Fabry disease. Ries M, Mengel E, Kutschke G, Kim KS, Birklein F, Krummenauer F, Beck M.J Inherit Metab Dis. 2003;26(4):413-4.

60. Impaired small fiber conduction in patients with Fabry disease: a neurophysiological case-control study. Üçeyler N, Kahn AK, Kramer D, Zeller D, Casanova-Molla J, Wanner C, Weidemann F, Katsarava Z, Sommer C.BMC Neurol. 2013 May 24;13:47

61. Changes in Ionic Conductance Signature of Nociceptive Neurons Underlying Fabry Disease Phenotype. Namer B, Ørstavik K, Schmidt R, Mair N, Kleggetveit IP, Zeidler M, Martha T, Jorum E, Schmelz M, Kalpachidou T, Kress M, Langeslag M.Front Neurol. 2017 Jul 14;8:335

62. Small fiber dysfunction predominates in Fabry neuropathy. Dütsch M, Marthol H, Stemper B, Brys M, Haendl T, Hilz MJ.J Clin Neurophysiol. 2002 Dec;19(6):575-86.

63. Autonomic skin responses in females with Fabry disease. Møller AT, Bach FW, Feldt-Rasmussen U, Rasmussen AK, Hasholt L, Sommer C, Kølvraa S, Jensen TS.J Peripher Nerv Syst. 2009 Sep;14(3):159-64

64. Involvement of peripheral nerve and muscle in Fabry's disease. Histologic, ultrastructural, and morphometric studies. Sima AA, Robertson DM.Arch Neurol. 1978 May;35(5):291-301.

65. Small-fibre neuropathy in female Fabry patients: reduced allodynia and skin blood flow after topical capsaicin. Moller AT, Feldt-Rasmussen U, Rasmussen AK, Sommer C, Hasholt L, Bach FW, Kølvraa S, Jensen TS.J Peripher Nerv Syst. 2006 Jun;11(2):119-25

66. Restless legs syndrome in Fabry disease: clinical feature associated to neuropathic pain is overlooked. Domínguez RO, Michref A, Tanus E, Amartino H.Rev Neurol. 2007 Oct 16-31;45(8):474-8

67. Neuropathic pain in Fabry's disease: heterogeneous remission in three years of enzyme replacement therapy. Domínguez RO, Amartino H, Chamoles NA; Grupo de Estudio de la Enfermedad de Fabry.Rev Neurol. 2006 Aug 16-31;43(4):201-6.

68. Clinical manifestations of Fabry disease in children: data from the FabryOutcome Survey. Ramaswami U, Whybra C, Parini R, Pintos-Morell G, Mehta A, Sunder-Plassmann G, Widmer U, Beck M; FOS European Investigators.Acta Paediatr. 2006 Jan;95(1):86-92

69. Later-onset Fabry disease: an adult variant presenting with the cramp-fasciculation syndrome. Nance CS, Klein CJ, Banikazemi M, Dikman SH, Phelps RG, McArthur JC, Rodriguez M, Desnick RJ.Arch Neurol. 2006 Mar;63(3):453-7

70. Small fiber neuropathy in female patients with fabry disease. Liguori R, Di Stasi V, Bugiardini E, Mignani R, Burlina A, Borsini W, Baruzzi A, Montagna P, Donadio V.Muscle Nerve. 2010 Mar;41(3):409-12

71. Loss of small peripheral sensory neurons in Fabry disease. Histologic and morphometric evaluation of cutaneous nerves, spinal ganglia, and posterior columns. Onishi A, Dyck PJ.Arch Neurol. 1974 Aug;31(2):120-7

72. Corneal confocal microscopy: a novel noninvasive means to diagnose neuropathy in patients with Fabry disease. Tavakoli M, Marshall A, Thompson L, Kenny M, Waldek S, Efron N, Malik RA.Muscle Nerve. 2009 Dec;40(6):976-84

73. Generalized anhidrosis associated with Fabry's disease. Kang WH, Chun SI, Lee S.J Am Acad Dermatol. 1987 Nov;17(5 Pt 2):883-7.

74. Restoration of peripheral neuropathy in Fabry mice via intrathecal administration of an adeno-associated virus vector encoding mGLA cDNA. Higuchi T, Shimada Y, Takahashi Y, Kato F, Ohashi T, Kobayashi H.Mol Genet Metab. 2024 Jul 27;143(1-2):108545.

75. Detection of a characteristic painful neuropathy in Fabry disease: a pilot study. Maag R, Binder A, Maier C, Scherens A, Toelle T, Treede RD, Baron R.Pain Med. 2008 Nov;9(8):1217-23.

76. Fabry's disease on the mechanism of the peripheral nerve involvement. Fukuhara N, Suzuki M, Fujita N, Tsubaki T.Acta Neuropathol. 1975 Oct 27;33(1):9-21

77. Carbamazepine in Fabry's disease: effective analgesia with dose-dependent exacerbation of autonomic dysfunction.Filling-Katz MR, Merrick HF, Fink JK, Miles RB, Sokol J, Barton NW.Neurology. 1989 Apr;39(4):598-600

78. The anatomical substratum of pain evidence derived from morphometric studies on peripheral nerve. Thomas PK.Can J Neurol Sci. 1974 May;1(2):92-7

79. Ultrastructure of muscle and sensory nerve in Fabry's disease. Tomé FM, Fardeau M, Lenoir G.Acta Neuropathol. 1977 Jun 15;38(3):187-94

80. Prevalence of Fabry disease in patients with chronic pain: Lessons from the DOUFAB and DOUFABIS studies. Angelini C, Bar C, Baudier MP, Fergelot P, Lancelot G, Rooryck C, Germain DP, Jabbour F, Blanchet AS, Cauchie A, Sarrazin E, Bellance R, Lefaucheur JP, Bismuth J, Ranque-Garnier S, Corand V, Coupry I, Goizet C; DOUFABIS Consortium.Eur J Pain. 2024 Aug 4. doi: 10.1002/ejp.4708

81. Depression, sleep disturbances, pain, disability and quality of LIFE in Brazilian Fabry disease patients. Rosa Neto NS, Bento JCB, Pereira RMR.Mol Genet Metab Rep. 2019 Dec 2;22:10054

82. Corneal confocal microscopy detects corneal nerve damage and increased dendritic cells in Fabry disease. Bitirgen G, Turkmen K, Malik RA, Ozkagnici A, Zengin N.Sci Rep. 2018 Aug 16;8(1):12244.
83. Pain in Fabry Disease: Could Spinal Cord Stimulation be a Solution? Buonanno P, Capuano I, Riccio E, Pisani A.Indian J Nephrol. 2023 Sep-Oct;33(5):394.
84. Gastrointestinal symptoms and delayed gastric emptying in Fabry's disease: response to metoclopramide. Argoff CE, Barton NW, Brady RO, Ziessman HA.Nucl Med Commun. 1998 Sep;19(9):887-91
85. Gastrointestinal Manifestations and Treatment Options in Fabry Disease Patients. A Systematic Review. Radulescu D, Crisan D, Militaru V, Buzdugan E, Stoicescu L, Grosu A, Vlad C, Grapa C, Radulescu ML.J Gastrointestin Liver Dis. 2022 Mar 19;31(1):98-106
86. Non-specific gastrointestinal features: Could it be Fabry disease? Hilz MJ, Arbustini E, Dagna L, Gasbarrini A, Goizet C, Lacombe D, Liguori R, Manna R, Politei J, Spada M, Burlina A.Dig Liver Dis. 2018 May;50(5):429-437.
87. Gastrointestinal Involvement in Anderson-Fabry Disease: A Narrative Review. Caputo F, Lungaro L, Galdi A, Zoli E, Giancola F, Caio G, De Giorgio R, Zoli G.Int J Environ Res Public Health. 2021 Mar 23;18(6):3320.
88. Fabry disease - a multisystemic disease with gastrointestinal manifestations. Lenders M, Brand E.Gut Microbes. 2022 Jan-Dec;14(1):2027852.
89. Understanding the gastrointestinal manifestations of Fabry disease: promoting prompt diagnosis. Zar-Kessler C, Karaa A, Sims KB, Clarke V, Kuo B.Therap Adv Gastroenterol. 2016 Jul;9(4):626-34.
90. Gastrointestinal symptoms in Fabry disease: everything is possible, including treatment. Hoffmann B, Keshav S.Acta Paediatr. 2007 Apr;96(455):84-6.
91. Gastrointestinal involvement in Fabry disease. So important, yet often neglected. Politei J, Thurberg BL, Wallace E, Warnock D, Serebrinsky G, Durand C, Schenone AB.Clin Genet. 2016 Jan;89(1):5-9
92. Gastrointestinal symptoms in 342 patients with Fabry disease: prevalence and response to enzyme replacement therapy. Hoffmann B, Schwarz M, Mehta A, Keshav S; Fabry Outcome Survey European Investigators.Clin Gastroenterol Hepatol. 2007 Dec;5(12):1447-53
93. Assessment of small fiber neuropathy in patients carrying the non-classical Fabry variant p.D313Y. von Cossel K, Muschol N, Friedrich RE, Glatzel M, Ammer L, Lohmöller B, Bendszus M, Mautner VF, Godel T.Muscle Nerve. 2021 May;63(5):745-750
94. Gastrointestinal manifestations of Fabry disease: clinical response to enzyme replacement therapy. Banikazemi M, Ullman T, Desnick RJ.Mol Genet Metab. 2005 Aug;85(4):255-9.
95. Abdominal pain and chronic diarrhea in a 55-year-old woman. Schiller D, Schöfl R, Mahévas M.Rev Med Interne. 2015 Feb;36(2):135-9
96. Gastrointestinal phenotype of fabry disease in a patient with pseudoobstruction syndrome. Buda P, Wieteska-Klimczak A, Ksiazyk J, Gietka P, Smorczewska-Kiljan A, Pronicki M, Czartoryska B, Tylki-Szymanska A.JIMD Rep. 2012;4:25-8.
97. Improvement of gastrointestinal symptoms in a significant proportion of male patients with classic Fabry disease treated with agalsidase beta: A FabryRegistry analysis stratified by phenotype. Hopkin RJ, Feldt-Rasmussen U, Germain DP, Jovanovic A, Martins AM, Nicholls K, Ortiz A, Politei J, Ponce E, Varas C, Weidemann F, Yang M, Wilcox WR.Mol Genet Metab Rep. 2020 Oct 30;25:100670.

98. Improvement of Fabry Disease-Related Gastrointestinal Symptoms in a Significant Proportion of Female Patients Treated with Agalsidase Beta: Data from the Fabry Registry. Wilcox WR, Feldt-Rasmussen U, Martins AM, Ortiz A, Lemay RM, Jovanovic A, Germain DP, Varas C, Nicholls K, Weidemann F, Hopkin RJ.JIMD Rep. 2018;38:45-51

99. Small bowel ischaemia in Fabry's disease. Jardine DL, Fitzpatrick MA, Troughton WD, Tie AB.J Gastroenterol Hepatol. 1994 Mar-Apr;9(2):201-4

100. Relief of gastrointestinal symptoms under enzyme replacement therapy [corrected] in patients with Fabry disease. Dehout F, Roland D, Treille de Granseigne S, Guillaume B, Van Maldergem L.J Inherit Metab Dis. 2004;27(4):499-505.

101. Effect of enzyme-replacement therapy on gastrointestinal symptoms in Fabrydisease. Hoffmann B, Reinhardt D, Koletzko B.Eur J Gastroenterol Hepatol. 2004 Oct;16(10):1067-9

102. Altered globotriaosylceramide accumulation and mucosal neuronal fiber density in the colon of the Fabry disease mouse model. Masotti M, Delprete C, Dothel G, Donadio V, Rimondini R, Politei JM, Liguori R, Caprini M.Neurogastroenterol Motil. 2019 Mar;31(3):e13529

103. Gastrointestinal Sensory Neuropathy and Dysmotility in Fabry Disease: Presentations and Effect on Patient's Quality of Life. Bar N, Karaa A, Kiser K, Kuo B, Zar-Kessler C.Clin Transl Gastroenterol. 2023 Dec 1;14(12):e00633

104. Gastrointestinal Symptoms of Patients with Fabry Disease. Pensabene L, Sestito S, Nicoletti A, Graziano F, Strisciuglio P, Concolino D.Gastroenterol Res Pract. 2016;2016:9712831.

105. Pathologic substrate of gastropathy in Anderson-Fabry disease. Di Toro A, Narula N, Giuliani L, Concardi M, Smirnova A, Favalli V, Urtis M, Alvisi C, Antoniazzi E, Arbustini E.Orphanet J Rare Dis. 2020 Jun 22;15(1):156

106. Pathophysiologic and ultrastructural basis for intestinal symptoms in Fabry'sdisease. O'Brien BD, Shnitka TK, McDougall R, Walker K, Costopoulos L, Lentle B, Anholt L, Freeman H, Thomson AB.Gastroenterology. 1982 May;82(5 Pt 1):957-62

Digital Health Technology(ies) (DHTs) in Fabry disease

A comprehensive literature search was conducted to identify relevant studies on DHTs findings in Fabry disease. The search strategy utilized Medical Subject Headings (MeSH) and keyword terms to ensure a broad yet targeted selection of studies.

Fabry disease was searched using MeSH terms and synonyms, including "Fabry Disease" and "Anderson-Fabry Disease." The second component included DHTs-related terms such as "digital*" "technology" "APP" "artificial intelligence", “device*” and "biosensor" These terms were combined using Boolean operators (*AND*, *OR*) to refine the search strategy and retrieve relevant studies.

PRISMA

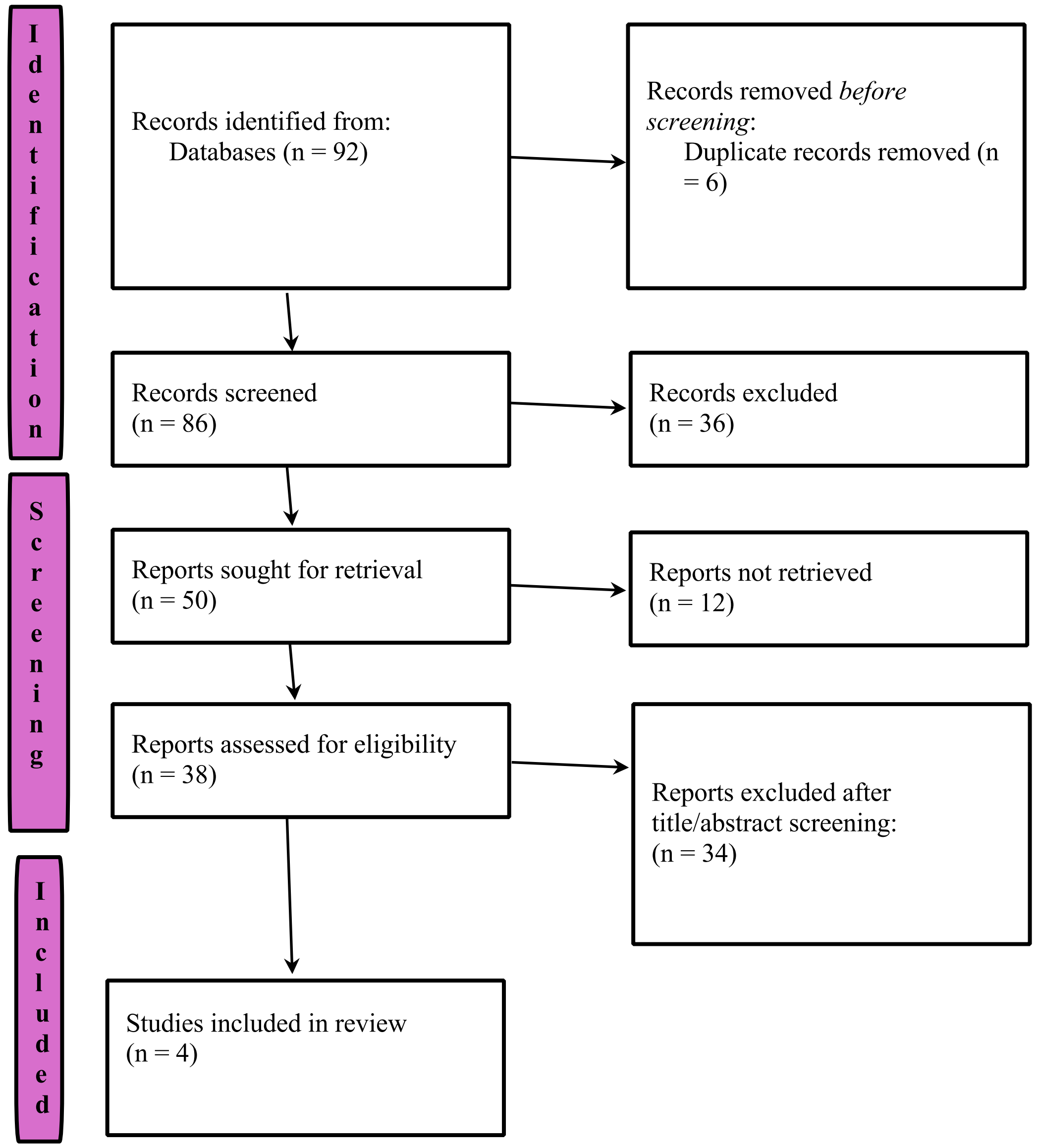

References:

1. Deep learning assisted retinal microvasculature assessment and cerebral small vessel disease in Fabry disease. Li Y, Zhou X, Li J, Zhao Y, Yuan Y, Yang B, Xu J, Wei Q, Yan X, Zhang W, Wu Y. Orphanet J Rare Dis. 2025 Apr 3;20(1):158. doi: 10.1186/s13023-025-03627-1.
2. A new approach to identifying patients with elevated risk for Fabry disease using a machine learning algorithm.Jefferies JL, Spencer AK, Lau HA, Nelson MW, Giuliano JD, Zabinski JW, Boussios C, Curhan G, Gliklich RE, Warnock DG.Orphanet J Rare Dis. 2021 Dec 20;16(1):518. doi: 10.1186/s13023-021-02150-3.
3. Computer-assisted retinal vessel diameter evaluation in Fabry disease.Sodi A, Nicolosi C, Vicini G, Lenzetti C, Virgili G, Rizzo S. Eur J Ophthalmol. 2021 Jan;31(1):173-178. doi: 10.1177/1120672119886985.
4. Fabry App: the value of a portable technology in recording day-to-day patient monitored information in patients with Fabry disease. Orphanet J Rare Dis. 2024 Jan 11;19(1):13. doi: 10.1186/s13023-023-02999-6.
