# Supplementary material for: Neurological management and outcome measures in Fabry disease: consensus statements from the Italian Fabry disease neurological working group
Source: Orphanet J Rare Dis. 2026 Apr 24;21:222. doi: 10.1186/s13023-026-04361-y (PMC13277257; doi:10.1186/s13023-026-04361-y)
Supplement: Supplementary file 2 — Supplementary Material 2 [file 13023_2026_4361_MOESM2_ESM.pdf]

CONSENSUS FABRY 1st Survey

First Survey

In the first Round you will start by answering a Survey with graded answers where you have the possibility to choose from 5 grades:
1.Strongly Disagree, 2.Disagree, 3.Neither agree or disagree, 4.Agree, 5.Strongly Agree.
All statements are mandatory. If you do not know how to answer please select 3.Neither agree or disagree

You can always check the literature search strategy at: https://drive.google.com/drive/folders/1aMuax1FSyNxEbI9sknlPF5heal_sXLZ9?usp=sharing

CONSENSUS FABRY 1st Survey

NEUROIMAGING STATEMENTS

Please now vote the Neuroimaging statements

* 1. Brain MRI with a standardized protocol, including T1-weighted, T2-weighted, FLAIR, diffusion-weighted imaging (DWI), and T2* weighted Gradient Echo (GRE) or, if available, susceptibility-weighted imaging (SWI), should be the primary modality for the diagnosis and follow-up of neuroradiological involvement in Fabry disease

- ◯ Strongly diagree
- ◯ Disagree
- ◯ Neither agree nor disagree
- ◯ Agree
- ◯ Strongly Agree

* 2. Magnetic Resonance Angiography should be performed in all Fabry patients to detect vertebrobasilar dolichoectasia and other cerebrovascular abnormalities.

- ◯ Strongly diagree
- ◯ Disagree
- ◯ Neither agree nor disagree
- ◯ Agree
- ◯ Strongly Agree

* 3. Patients with Fabry disease should undergo brain MRI at baseline and every 2–3 years to monitor cerebrovascular involvement, with more frequent imaging in those with rapid clinical progression or new neurological symptoms.

- ◯ Strongly diagree
- ◯ Disagree
- ◯ Neither agree nor disagree
- ◯ Agree
- ◯ Strongly Agree

* 4. In patients with contraindications to MRI (e.g., implantable cardioverter-defibrillators), brain CT may be considered as an alternative for detecting gross structural abnormalities and ischemic lesions. However, CT has limited sensitivity for Fabry-related microvascular pathology, and routine periodic scanning is not recommended in the absence of significant clinical changes.

- ◯ Strongly disagree
- ◯ Disagree
- ◯ Neither agree nor disagree
- ◯ Agree
- ◯ Strongly Agree

* 5. The assessment of cerebrovascular involvement in Fabry disease should adhere to standardized criteria to ensure consistency in diagnosis, disease monitoring, and research. The STRIVE (Standards for Reporting Vascular Changes on Neuroimaging) criteria should serve as a reference framework for evaluating small vessel disease-related changes.

- ◯ Strongly disagree
- ◯ Disagree
- ◯ Neither agree nor disagree
- ◯ Agree
- ◯ Strongly Agree

* 6. The following neuroimaging biomarkers and rating scales should be systematically used:
-White Matter Hyperintensities (WMH): Assessed using the Fazekas Scale to quantify periventricular and deep white matter lesions.
-Lacunes: Defined by STRIVE criteria (3–15 mm subcortical infarcts) and recorded based on number, location, and volume.
-Cerebral Microbleeds (CMBs): Evaluated using the Microbleed Anatomical Rating Scale (MARS) on SWI/GRE sequences.
-Enlarged Perivascular Spaces (EPVS): Quantified using the EPVS Scale, separately for the centrum semiovale and basal ganglia.
-Brain Atrophy: Monitored using the Global Cortical Atrophy (GCA) scale for visual assessment.
-Pulvinar Sign: Systematically reported on T1-weighted MRI, given its potential Fabry-specific significance.
-Basilar Dolichoectasia: Assessed using the Smoker Criteria or Powers Criteria on MRA, documenting vertebrobasilar artery elongation and dilation.

- ◯ Strongly disagree
- ◯ Disagree
- ◯ Neither agree nor disagree
- ◯ Agree
- ◯ Strongly Agree

* 7. Advanced neuroimaging techniques such as perfusion MRI, diffusion tensor imaging (DTI), and functional MRI (fMRI) have shown potential for detecting early microstructural and functional alterations in Fabry disease. However, their routine use in clinical practice is currently limited by availability, cost, and standardization issues.

- ◯ Strongly disagree
- ◯ Disagree
- ◯ Neither agree nor disagree
- ◯ Agree
- ◯ Strongly Agree

* 8. Transcranial Doppler ultrasound may provide valuable information on cerebral hemodynamics and microvascular dysfunction in Fabry disease, but its routine use in clinical practice remains uncertain. It may be beneficial for selected patients, particularly for assessing cerebrovascular reactivity.

- ◯ Strongly disagree
- ◯ Disagree
- ◯ Neither agree nor disagree
- ◯ Agree
- ◯ Strongly Agree

* 9. While PET and SPECT imaging may provide insights into metabolic and functional brain changes in Fabry disease, their role in routine clinical practice remains limited. These modalities may be useful in research settings or selected cases with atypical presentations or suspected neurodegeneration.

- Strongly disagree
- Disagree
- Neither agree nor disagree
- Agree
- Strongly Agree

CONSENSUS FABRY 1st Survey

Cerebrovascular and cognitive impairment

All statements are mandatory

* 10. Cognitive screening should be performed in all Fabry disease patients at diagnosis, regardless of symptom presentation.

- Strongly diagree
- Disagree
- Neither agree nor disagree
- Agree
- Strongly Agree

* 11. The Montreal Cognitive Assessment (MoCA) should be preferred over the Mini-Mental State Examination (MMSE) due to its higher sensitivity in detecting mild cognitive impairment.

- Strongly diagree
- Disagree
- Neither agree nor disagree
- Agree
- Strongly Agree

* 12. A comprehensive neuropsychological evaluation should be performed in Fabry patients with suspected cognitive decline, including assessments of executive function, attention, and processing speed.

- Strongly diagree
- Disagree
- Neither agree nor disagree
- Agree
- Strongly Agree

* 13. Depression, anxiety, and other mood disorders should be routinely screened in Fabry patients with cognitive complaints, as they may contribute to cognitive symptoms.

- Strongly diagree
- Disagree
- Neither agree nor disagree
- Agree
- Strongly Agree

* 14. The Hospital Anxiety and Depression scale (HADS) should be used to assess mood disorders in Fabry patients

- Strongly diagree
- Disagree
- Neither agree nor disagree
- Agree
- Strongly Agree

* 15. Cognitive function should be reassessed every 2 years in asymptomatic patients and every 12 months in patients with prior stroke, progressive symptoms, or neurological involvement.

- Strongly diagree
- Disagree
- Neither agree nor disagree
- Agree
- Strongly Agree

* 16. Cognitive rehabilitation programs should be considered for patients with executive dysfunction, attention deficits, or processing speed impairments, particularly those affecting daily life.

- Strongly diagree
- Disagree
- Neither agree nor disagree
- Agree
- Strongly Agree

* 17. Patients should be educated about stroke symptoms and the importance of early medical intervention in case of new neurological events.

- Strongly diagree
- Disagree
- Neither agree nor disagree
- Agree
- Strongly Agree

* 18. Fibrinolysis and/or thrombectomy are not contraindicated in Fabry disease

- Strongly diagree
- Disagree
- Neither agree nor disagree
- Agree
- Strongly Agree

* 19. Patients with Fabry disease and a history of stroke should undergo neurological follow-up at least annually, including clinical examination and, if appropriate, repeat neuroimaging.

- Strongly diagree
- Disagree
- Neither agree nor disagree
- Agree
- Strongly Agree

* 20. All FD patients with left ventricular hypertrophy (LVH), conduction abnormalities, or prior stroke/TIA should undergo prolonged ECG monitoring

- Strongly diagree
- Disagree
- Neither agree nor disagree
- Agree
- Strongly Agree

* 21. The Modified Rankin Scale (mRS) should be the primary scale used to assess long-term disability after stroke.

- Strongly diagree
- Disagree
- Neither agree nor disagree
- Agree
- Strongly Agree

* 22. Barthel Index (BI) and NIHSS may complement mRS in evaluating functional independence and stroke severity.

- Strongly diagree
- Disagree
- Neither agree nor disagree
- Agree
- Strongly Agree

CONSENSUS FABRY 1st Survey

Peripheral nervous system involvement (including gastrointestinal involvement)

All queries are mandatory

* 23. Clinicians should use validated disease-specific questionnaires for neuropathic pain assessment, such as the Würzburg Fabry Pain Questionnaire for adults and the Fabry-specific Pediatric Health and Pain Questionnaire for children.

- Strongly diagree
- Disagree
- Neither agree nor disagree
- Agree
- Strongly Agree

* 24. Bedside sensory tests, comprising FabryScan assessment, should be incorporated into routine evaluations to detect small fiber neuropathy.

- Strongly diagree
- Disagree
- Neither agree nor disagree
- Agree
- Strongly Agree

* 25. For patients with atypical presentations or unclear clinical findings, Quantitative Sensory Testing (QST) and skin punch biopsy for intra-epidermal nerve fiber density may be considered to confirm small fiber involvement but should not be mandatory. Skin biopsy is recommended only in selected cases.

- Strongly diagree
- Disagree
- Neither agree nor disagree
- Agree
- Strongly Agree

* 26. Sudoscan, which measures ESC, may be used as a non-invasive screening tool for sudomotor dysfunction but should complement rather than replace standard diagnostic tests such as QST or skin biopsy.

- Strongly diagree
- Disagree
- Neither agree nor disagree
- Agree
- Strongly Agree

* 27. In addition to the Würzburg Fabry Pain Questionnaire,the Brief Pain Inventory (BPI), Short-Form McGill Pain Questionnaire (SF-MPQ), and Neuropathic Pain Symptom Inventory may be used for a broader pain assessment.

- ◯ Strongly diagree
- ◯ Disagree
- ◯ Neither agree nor disagree
- ◯ Agree
- ◯ Strongly Agree

* 28. Pain questionnaires should be administered at diagnosis, every 6–12 months, and whenever therapy modifications occur. Sensory testing should be repeated annually or in response to symptom progression.

- ◯ Strongly diagree
- ◯ Disagree
- ◯ Neither agree nor disagree
- ◯ Agree
- ◯ Strongly Agree

* 29. Skin biopsy should be performed in cases of atypical symptom presentation or when confirmation of small fiber neuropathy is necessary. Routine biopsy is not required for all patients, but a baseline biopsy can be useful for documenting nerve fiber density.

- ◯ Strongly diagree
- ◯ Disagree
- ◯ Neither agree nor disagree
- ◯ Agree
- ◯ Strongly Agree

* 30. A comprehensive neurological assessment, including FabryScan, should be performed annually to monitor disease progression.

- ◯ Strongly diagree
- ◯ Disagree
- ◯ Neither agree nor disagree
- ◯ Agree
- ◯ Strongly Agree

* 31. Autonomic dysfunction assessment should be tailored to the patient’s symptoms, with regular monitoring in those experiencing progressive small fiber neuropathy, cardiovascular dysautonomia, or severe gastrointestinal symptoms.

- ◯ Strongly diagree
- ◯ Disagree
- ◯ Neither agree nor disagree
- ◯ Agree
- ◯ Strongly Agree

* 32. Electroneurography is not recommended in Fabry disease unless large-fiber involvement is suspected.

- Strongly diagree
- Disagree
- Neither agree nor disagree
- Agree
- Strongly Agree

* 33. Gastrointestinal dysmotility requires early evaluation using validated clinical scales and motility tests to optimize symptom management and improve patient quality of life.

- Strongly diagree
- Disagree
- Neither agree nor disagree
- Agree
- Strongly Agree

* 34. The administration of COMPASS31 questionnaire is useful in overall assessment of the patient’s dysautonomia

- Strongly diagree
- Disagree
- Neither agree nor disagree
- Agree
- Strongly Agree

* 35. The 24-hour or 7-day Fabry disease Patient-Reported Outcome-Gastrointestinal (FABPRO-GI) questionnaire should be used to evaluate bloating, abdominal pain, diarrhoea, constipation, and reflux.

- Strongly diagree
- Disagree
- Neither agree nor disagree
- Agree
- Strongly Agree

* 36. The FABPRO-GI questionnaire should be administered at diagnosis, regardless of symptom severity, and repeated every 6-12 months to track symptom progression or treatment response.

- Strongly diagree
- Disagree
- Neither agree nor disagree
- Agree
- Strongly Agree

* 37. Nutritional status (BMI, albumin) should be monitored annually, especially in pediatric males with growth concerns.

- Strongly diagree
- Disagree
- Neither agree nor disagree
- Agree
- Strongly Agree

* 38. 5Q-5D-5L should be the primary tool for measuring generic health-related QoL across mobility, self-care, pain/discomfort, and psychological well-being and should be administered annually to track longitudinal changes, with additional assessments during major treatment transitions.

- Strongly diagree
- Disagree
- Neither agree nor disagree
- Agree
- Strongly Agree

* 39. The FD-PRO is a validated, disease-specific patient-reported outcome instrument designed to measure symptom severity in Fabry disease which assesses neuropathic pain, gastrointestinal symptoms, autonomic dysfunction, and overall disease burden. In clinical practice, it should be used at baseline, every 6–12 months, and during therapy modifications to track disease progression and treatment efficacy.

- Strongly diagree
- Disagree
- Neither agree nor disagree
- Agree
- Strongly Agree

CONSENSUS FABRY 1st Survey

Digital tools

All queries are mandatory

* 40. There are no validated digital tools for Fabry disease assessment to date, with no pivotal studies on this topic published. More studies need to be done to further develop and validate such digital tools in Fabry disease.

- Strongly diagree
- Disagree
- Neither agree nor disagree
- Agree
- Strongly Agree
