# Supplementary material for: Neurological management and outcome measures in Fabry disease: consensus statements from the Italian Fabry disease neurological working group
Source: Orphanet J Rare Dis. 2026 Apr 24;21:222. doi: 10.1186/s13023-026-04361-y (PMC13277257; doi:10.1186/s13023-026-04361-y)
Supplement: Supplementary file 3 — Supplementary Material 3 [file 13023_2026_4361_MOESM3_ESM.pdf]

**SUPPLEMENTARY FILE 3**

# CENTRAL NERVOUS SYSTEM: CONSENSUS STATEMENTS

| STATEMENT | % of experts voting *agree* or *strong agree* | |
|---|---|---|
| Brain MRI with a standardized protocol, including T1-weighted, T2-weighted, FLAIR, diffusion-weighted imaging (DWI), and T2* weighted Gradient Echo (GRE) or, if available, susceptibility-weighted imaging (SWI), should be the primary modality for the diagnosis and follow-up of neuroradiological involvement in Fabry disease | **93,75%**<br>**Mean 4.25** | **STRONG CONSENSUS** |
| Magnetic Resonance Angiography should be performed in all Fabry patients to detect vertebrobasilar dolichoectasia and other cerebrovascular abnormalities at the baseline | **85.71%**<br>**Mean 4.36** | **STRONG CONSENSUS** |
| Patients with Fabry disease should undergo brain MRI at baseline and every 3 years to monitor cerebrovascular involvement, with more frequent imaging in those with clinical progression or new neurological symptoms | **92.85%**<br>**Mean 4.64** | **STRONG CONSENSUS** |
| In patients with contraindications to MRI (e.g., implantable cardioverter-defibrillators), brain CT may be considered as an alternative for detecting gross structural abnormalities and ischemic lesions. However, CT has limited sensitivity for Fabry- related microvascular pathology, and routine periodic scanning is not recommended in the absence of significant clinical changes. | **100%**<br>**Mean 4.63** | **STRONG CONSENSUS** |
| The assessment of cerebrovascular involvement in Fabry disease should adhere to standardized criteria to ensure consistency in diagnosis, disease monitoring, and research. The STRIVE (Standards for Reporting Vascular Changes on Neuroimaging) criteria should serve as a reference framework for evaluating small vessel disease-related changes. | **100%**<br>**Mean 4.38** | **STRONG CONSENSUS** |
| Exploratory (for research only):The following neuroimaging biomarkers and rating scales should be systematically used:-White Matter Hyperintensities (WMH): Assessed using the Fazekas Scale to quantify periventricular and deep white matter lesions.-Lacunes: Defined by STRIVE criteria (3–15 mm subcortical infarcts) and recorded based on number, location, and volume.-Cerebral Microbleeds (CMBs): Evaluated using the Microbleed Anatomical Rating Scale (MARS) on SWI/GRE sequences.-Enlarged Perivascular Spaces (EPVS): Quantified using the EPVS Scale, separately for the centrum semiovale and basal ganglia.-Brain Atrophy: Monitored using the Global Cortical Atrophy (GCA) scale for visual assessment.-Pulvinar Sign: Systematically reported on T1-weighted MRI, given its potential Fabry-specific significance.-Basilar Dolichoectasia: Assessed using the Smoker Criteria or Powers Criteria on MRA, documenting vertebrobasilar artery elongation and dilation | **85.72%**<br>**Mean 4.14** | **STRONG CONSENSUS** |
| Advanced neuroimaging techniques such as perfusion MRI, diffusion tensor imaging (DTI), and functional MRI (fMRI) have shown potential for detecting early microstructural and functional alterations in Fabry disease. However, their routine use in clinical practice is currently limited by availability, cost, and standardization issues. | **75%**<br>**Mean 3.94** | **CONSENSUS** |
| Transcranial Doppler ultrasound may provide valuable information on cerebral hemodynamic and microvascular dysfunction in Fabry disease, but its routine use in clinical practice remains uncertain. It may be beneficial for selected patients, particularly for assessing cerebrovascular reactivity. | **75%**<br>**Mean 4** | **STRONG CONSENSUS** |
| While PET and SPECT imaging may provide insights into metabolic and functional brain changes in Fabry disease, their role in routine clinical practice remains limited. These modalities may be useful in research settings or selected cases with atypical presentations or suspected neurodegeneration. | **100%**<br>**Mean 4.44** | **STRONG CONSENSUS** |
| Cognitive screening should be performed in all Fabry disease patients at diagnosis, regardless of symptom presentation. | **100%**<br>**Mean 4.56** | **STRONG CONSENSUS** |
| Sleep disturbances may represent a significant concern in Fabry disease and should be actively investigated when suspected | **92.85%**<br>**Mean 4,36** | **STRONG CONSENSUS** |
| The Montreal Cognitive Assessment (MoCA) should be preferred over the Mini-Mental State Examination (MMSE) due to its higher sensitivity in detecting mild cognitive impairment. | **100%**<br>**Mean 4.50** | **STRONG CONSENSUS** |
| A comprehensive neuropsychological evaluation should be performed in Fabry patients with suspected cognitive decline, including assessments of executive function, attention, and processing speed. | **93.75%**<br>**Mean 4.44** | **STRONG CONSENSUS** |

**SUPPLEMENTARY FILE 3**

| | | |
|---|---|---|
| Depression, anxiety, and other mood disorders should be routinely screened in Fabry patients with cognitive complaints, as they may contribute to cognitive symptoms. | **100%**<br>**Mean 4.44** | **STRONG CONSENSUS** |
| The Hospital Anxiety and Depression scale (HADS) should be used to assess mood disorders in Fabry patients | **93.47%**<br>**Mean 4.57** | **STRONG CONSENSUS** |
| Cognitive function should be reassessed every 2 years in asymptomatic patients and every 12 months in patients with prior stroke, progressive symptoms, or neurological involvement. | **81.25%**<br>**Mean 4.06** | **STRONG CONSENSUS** |
| Cognitive rehabilitation programs should be considered for patients with executive dysfunction, attention deficits, or processing speed impairments, particularly those affecting daily life. | **75%**<br>**Mean 4.06** | **STRONG CONSENSUS** |
| Patients should be educated about stroke symptoms and the importance of early medical intervention in case of new neurological events. | **93.75**<br>**Mean 4.56** | **STRONG CONSENSUS** |
| Fibrinolysis and/or thrombectomy are not contraindicated in Fabry disease | **81.25%**<br>**Mean 4.19** | **STRONG CONSENSUS** |
| Patients with Fabry disease and a history of stroke should undergo neurological follow-up at least annually, including clinical examination and, if appropriate, repeat neuroimaging. | **81,25%**<br>**Mean 4.25** | **STRONG CONSENSUS** |
| All FD patients with left ventricular hypertrophy (LVH), conduction abnormalities, or prior stroke/TIA should undergo prolonged ECG monitoring | **75%**<br>**Mean 4.12** | **STRONG CONSENSUS** |
| The vascular risk factor profile of patients with Fabry disease should be evaluated and treated when indicated. Lifestyle changes such as stopping smoking, healthy diet and physical exercise should be advised. | **100%**<br>**Mean 4.64** | **STRONG CONSENSUS** |
| Secondary prevention for cerebrovascular disease in Fabry should adhere to the international guidelines (i.e. ESO and AHA-ASA) | **100%**<br>**Mean 4.86** | **STRONG CONSENSUS** |
| Otovestibular symptoms should be investigated at the time of neurological evaluation | **100%**<br>**Mean 4.79** | **STRONG CONSENSUS** |
| Audiometric examination should be performed in patients with Fabry disease at baseline | **100%**<br>**Mean 4.79** | **STRONG CONSENSUS** |
| Audiometric examination should be repeated every 3 years, or whenever hearing impairment (or its worsening) occurs | **100%**<br>**Mean 4.36** | **STRONG CONSENSUS** |
| The Modified Rankin Scale (mRS) should be the primary scale used to assess long-term disability after stroke. | **93.75%**<br>**Mean 4.56** | **STRONG CONSENSUS** |
| Barthel Index (BI) and NIHSS may complement mRS in evaluating functional independence and stroke severity. | **81,25%**<br>**Mean 4.06** | **STRONG CONSENSUS** |
| The modified Fazekas scale should be included in the radiological report. | **92.86%**<br>**Mean 4.43** | STRONG CONSENSUS |
| Fatigue severity scale is a good scale to monitor fatigue in Fabry disease | **92.86**<br>**Mean 4.43** | **STRONG CONSENSUS** |

SUPPLEMENTARY FILE 3

# PERIPHERAL NERVOUS SYSTEM: CONSENSUS STATEMENTS

| **STATEMENT** | **% of experts voting *agree* or *strong agree*** | |
|---|---|---|
| Clinicians should use validated disease-specific questionnaires for neuropathic pain assessment, such as the Würzburg Fabry Pain Questionnaire for adults | **100%**<br>**Mean 5** | **STRONG CONSENSUS** |
| Bedside sensory tests, comprising FabryScan assessment, maybe incorporated into routine evaluations. | **100%**<br>**Mean 4.57** | **STRONG CONSENSUS** |
| Quantitative Sensory Testing (QST) may be considered to confirm small fiber involvement. | **100%**<br>**Mean 4.93** | **STRONG CONSENSUS** |
| Skin punch biopsy is indicated to confirm small fiber involvement | **92.86%**<br>**Mean 4.50** | **STRONG CONSENSUS** |
| Pain questionnaires should be administered at diagnosis, every 12 months, and whenever pain management therapy occur | **100%**<br>**Mean 4.93** | **STRONG CONSENSUS** |
| A comprehensive neurological assessment, including FabryScan, should be performed annually to monitor disease progression. | **100%**<br>**Mean 5** | **STRONG CONSENSUS** |
| Electroneurography is not routinely recommended in Fabry disease unless large-fiber involvement is suspected. | **100%**<br>**Mean 4.79** | **STRONG CONSENSUS** |

SUPPLEMENTARY FILE 3

# AUTONOMOUS NERVOUS SYSTEM: CONSENSUS STATEMENTS

| STATEMENT | % of experts voting *agree* or *strong agree* | |
|---|---|---|
| Sympathetic Skin Response, which measures Electrochemical Skin Conductance, may be used as a non-invasive test for sudomotor dysfunction | **78.57 Mean 4** | **STRONG CONSENSUS** |
| Skin punch biopsy is indicated to confirm sudomotor dysfunction | **92.85% Mean 4.57** | **STRONG CONSENSUS** |
| Skin punch biopsy is indicated to confirm small fiber involvement | **100% Mean 4.93** | **STRONG CONSENSUS** |
| Autonomic dysfunction assessment and management should be tailored to the patient's symptoms, especially in those with autonomic small fiber neuropathy (i.e. cardiovascular dysautonomia and/or severe gastrointestinal symptoms). | **100% Mean 4.57** | **STRONG CONSENSUS** |
| Gastrointestinal dysmotility requires early evaluation using validated clinical scales to optimize symptom management and improve patient quality of life | **93.75% Mean 4.57** | **STRONG CONSENSUS** |
| The administration of COMPASS31 questionnaire is useful in overall assessment of the patient's dysautonomia | **81,25% Mean 4** | **STRONG CONSENSUS** |
| The 24-hour or 7-day Fabry disease Patient-Reported Outcome-Gastrointestinal (FABPRO-GI) questionnaire should be used to evaluate bloating, abdominal pain, diarrhoea, constipation, and reflux. | **87.50% Mean 4** | **STRONG CONSENSUS** |
| 5Q-5D-5L should be the primary tool for measuring generic health-related QoL across mobility, self-care, pain/discomfort, and psychological well-being and should be administered annually to track longitudinal changes, with additional assessments during major treatment transitions | **81,25% Mean 3.88** | **CONSENSUS** |
| The FD-PRO is a validated, disease-specific patient-reported outcome instrument designed to measure symptom severity in Fabry disease which assesses neuropathic pain, gastrointestinal symptoms, autonomic dysfunction, and overall disease burden. In clinical practice, it should be used at baseline, every 12 months, and whenever therapy modification occurs | **92.86% Mean 4.44** | **STRONG CONSENSUS** |
| There are no validated digital tools for Fabry disease assessment to date, with no pivotal studies on this topic published. More studies need to be done to further develop and validate such digital tools in Fabry disease | **93.75% Mean 4.56** | **STRONG CONSENSUS** |

## SUPPLEMENTARY FILE 3

# STATEMENTS WITHOUT CONSENSUS

| | | |
|---|---|---|
| Sudoscan, which measures ESC, may be used as a non-invasive screening tool for sudomotor dysfunction but should complement rather than replace standard diagnostic tests such as QST or skin biopsy. | **62,50%**<br>**Mean 3.50** | **NO CONSENSUS** |
| Sudoscan, which measures ESC, should be used as a non-invasive test for sudomotor dysfunction. | **21.43%**<br>**Mean 2.4** | **NO CONSENSUS** |
| The FABPRO-GI questionnaire should be administered at diagnosis, regardless of symptom severity, and repeated every 6-12 months to track symptom progression or treatment response. | **68.75%** | **NO CONSENSUS** |
| Nutritional status (BMI, albumin) should be monitored annually, especially in paediatric males with growth concerns. | **56,25%**<br>**NO CONSENSUS** | **NO CONSENSUS** |
